# Reprogramming of 3′ Untranslated Regions of mRNAs by Alternative Polyadenylation in Generation of Pluripotent Stem Cells from Different Cell Types

**DOI:** 10.1371/journal.pone.0008419

**Published:** 2009-12-23

**Authors:** Zhe Ji, Bin Tian

**Affiliations:** Department of Biochemistry and Molecular Biology, Graduate School of Biomedical Sciences and New Jersey Medical School, University of Medicine and Dentistry of New Jersey, Newark, New Jersey, United States of America; Centre de Regulació Genòmica, Spain

## Abstract

**Background:**

The 3′ untranslated regions (3′UTRs) of mRNAs contain cis elements involved in post-transcriptional regulation of gene expression. Over half of all mammalian genes contain multiple polyadenylation sites that lead to different 3′UTRs for a gene. Studies have shown that the alternative polyadenylation (APA) pattern varies across tissues, and is dynamically regulated in proliferating or differentiating cells. Generation of induced pluripotent stem (iPS) cells, in which differentiated cells are reprogrammed to an embryonic stem (ES) cell-like state, has been intensively studied in recent years. However, it is not known how 3′UTRs are regulated during cell reprogramming.

**Methods/Main Findings:**

Using a computational method that robustly examines APA across DNA microarray data sets, we analyzed 3′UTR dynamics in generation of iPS cells from different cell types. We found that 3′UTRs shorten during reprogramming of somatic cells, the extent of which depends on the type of source cell. By contrast, reprogramming of spermatogonial cells involves 3′UTR lengthening. The alternative polyadenylation sites that are highly responsive to change of cell state in generation of iPS cells are also highly regulated during embryonic development in opposite directions. Compared with other sites, they are more conserved, can lead to longer alternative 3′UTRs, and are associated with more cis elements for polyadenylation. Consistently, reprogramming of somatic cells and germ cells involves significant upregulation and downregulation, respectively, of mRNAs encoding polyadenylation factors, and RNA processing is one of the most significantly regulated biological processes during cell reprogramming. Furthermore, genes containing target sites of ES cell-specific microRNAs (miRNAs) in different portions of 3′UTR are distinctively regulated during cell reprogramming, suggesting impact of APA on miRNA targeting.

**Conclusions/Significance:**

Taken together, these findings indicate that reprogramming of 3′UTRs by APA, which result from regulation of both general polyadenylation activity and cell type-specific factors and can reset post-transcriptional gene regulatory programs in the cell, is an integral part of iPS cell generation, and the APA pattern can be a good biomarker for cell type and state, useful for sample classification. The results also suggest that perturbation of the mRNA polyadenylation machinery or RNA processing activity may facilitate generation of iPS cells.

## Introduction

Embryonic stem (ES) cells, derived from inner cell mass of the blastocyst, an early stage embryo, are pluripotent cells which can differentiate into any of the three germ layers, and thus are believed to have the potential to treat a wide range of degenerative diseases and tissue damages [1]. Recent advances in induced pluripotent stem (iPS) cells, which are derived by reprogramming of differentiated cells to ES-like cells using a set of defined factors [2], [3], have stimulated the excitement that many ethical and technical barriers associated with clinical application of ES cells may be overcome by using iPS cells. Current approaches to generate iPS cells involve ectopic expression of a set of transcription factors that are essential for self-renewal, such as Oct4, Sox2, Klf4, c-Myc, Nanog, and LIN28, through various viral vectors, plasmids, or recombinant proteins [4], [5]. In addition, pluripotent ES-like cells have been derived from germ cells under proper culturing conditions, including primordial germ cells from the embryo [6] and spermatogonial cells from neonatal or adult testes [7], [8], [9].

The 3′ untranslated regions (3′UTRs) of mRNAs contain various cis elements involved in post-transcriptional gene regulation, such as mRNA localization, stability, and translation [10], [11], [12]. Cis elements that are widely encoded in 3′UTRs include miRNA target sites [13], AU-rich elements [14], and GU-rich elements [15]. Over half of all mammalian genes have multiple polyadenylation sites, or poly(A) sites, resulting in mRNA isoforms with different 3′UTRs and/or coding sequences (CDS) [16], [17]. Compared with constitutive regions of 3′UTRs, alternative regions are usually longer by ∼2 fold, have higher AU content, and contain more cis elements [18].

Poly(A) sites, recognized and processed by the mRNA polyadenylation machinery, are essential for 3′ end maturation of almost all mRNAs in eukaryotic cells [19], [20]. About 90 protein factors have been shown to be part of or associated with the mRNA polyadenylation machinery in human cells [21]. Both upstream and downstream elements surrounding a poly(A) site are critical for mRNA polyadenylation. For example, the CPSF complex interacts with the upstream AAUAAA/AUUAAA hexamer, also known as polyadenylation signal (PAS); and the CstF complex interacts with the downstream U-rich and GU-rich elements. In addition, various upstream and downstream auxiliary elements have been found to play regulatory roles in poly(A) site usage [22], [23].

Regulation of alternative polyadenylation (APA) under various biological conditions has been analyzed for many cellular and viral genes [24], [25]. Recent global analyses have indicated that the APA pattern varies among tissue types [26], [27]. For example, mRNAs expressed in brain tissues tend to have longer 3′UTRs than other tissue types [26], and those expressed in testes tend to have short 3′UTRs resulting from poly(A) sites that are not frequently used in other tissues [26], [28]. In addition, APA can be dynamically regulated in response to extracellular signals, for example activation of neuronal cells [29]. A general trend of 3′UTR shortening in proliferating cells was reported by Sandberg and coworkers [30]. Mayr and Bartel found that expression of mRNAs with shortened 3′UTRs are more apparent in transformed cells than nontransformed ones with similar proliferating rate [31]. We recently reported that 3′UTRs progressively lengthen via APA during mouse embryonic development [18] and this regulation coordinates with various aspects of development including proliferation, differentiation, and morphogenesis, and likely results from weakening of the general mRNA polyadenylation activity when cells are committed to specific types.

## Results

### Analysis of 3′UTR Regulation in Generation of iPS Cells Using a Robust Computational Method

A number of studies have used DNA microarrays to profile gene expression in reprogramming of somatic cells into iPS cells, all of which focused on mRNA levels. We were interested in how 3′UTRs were regulated during the reprogramming process. To this end, we collected data from a set of studies that utilized Affymetrix GeneChip microarrays, because their design was amenable to 3′UTR analysis for a large number of genes [18], [30]. As listed in Tables S1 and S2, these studies included 5 data sets for mouse iPS cells derived from B lymphocytes [32], mouse embryonic fibroblasts (MEFs) [33], and adult neural stem cells (NSCs) [34], [35], and 4 data sets of human iPS cells derived from neonatal foreskin fibroblasts and fetal lung fibroblasts [36], [37], [38], [39]. In addition, we included a data set for human pluripotent stem cells derived from spermatogonial cells (SC), a type of germ cell, from human adult testis [9]. For simplicity, these SC-derived cells were also referred to as iPS cells in this study.

**Figure 1:**
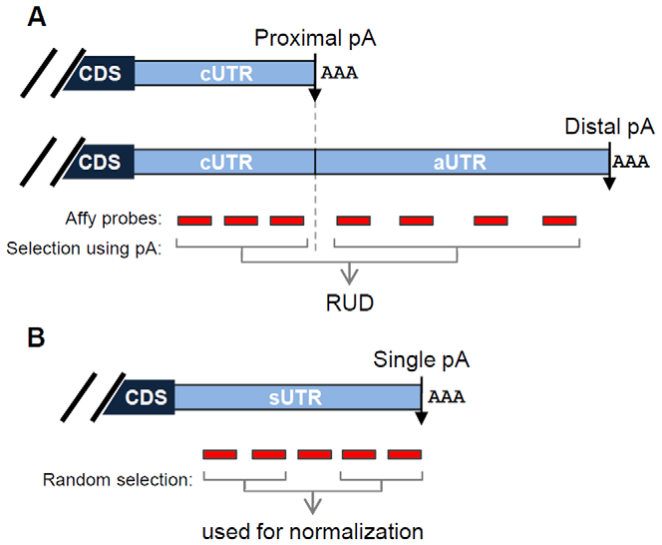
Schematic of APA and analysis of APA using Affymetrix GeneChip probes. (A) A hypothetical gene with 2 poly(A) sites in the 3′-most exon expresses 2 transcript isoforms with different 3′UTRs. The common region is named constitutive UTR (cUTR) and the alternative region alternative UTR (aUTR). The poly(A) sites are named proximal and distal poly(A) sites based on their locations relative to the coding sequence (CDS). Affymetrix (Affy) GeneChip probes targeting cUTRs and aUTRs are separated and compared to derive a Relative Usage of Distal poly(A) site (RUD) score. pA, poly(A) site; AAA, poly(A) tail. (B) A gene with a single poly(A) site expresses a transcript with a single 3′UTR, named sUTR. Affy probes targeting sUTRs were randomly selected, 2 probes from 5′ region and 2 from 3′ region, for normalizing RUD values (see Figure S1 for detail).

For genes with APA, the first and last poly(A) sites in the 3′-most exon were named proximal and distal sites, respectively (Figure 1). The regions upstream and downstream of a proximal site were named constitutive UTR (cUTR) and alternative UTR (aUTR), respectively. Accordingly, the 3′UTR in genes without APA was named single UTR (sUTR). Our method to analyze 3′UTR regulation was based on comparison of microarray probes targeting cUTRs with those targeting aUTRs with respect to intensity changes between samples in a sample set (Figures 1 and S1). A score named Relative Usage of Distal poly(A) site, or RUD, was used to represent relative 3′UTR length for a gene in a sample, with high RUD indicating long 3′UTR. The median RUD of all surveyed genes in a sample was used to represent global 3′UTR length for the sample.

Interestingly, we found that microarray samples processed at different times could have systematic differences in RUD (Figure S3), presumably due to differences in sample processing. We thus developed a method to normalize RUD values across samples by using probes targeting sUTRs (Figure S1). Our rationale was that comparing 5′ sUTR probes with 3′ ones (Figure 1B) could provide a background difference for probes in cUTRs and aUTRs, controlling variation of RUD values between samples that are attributable to technical reasons. As shown in Figure S3, this approach significantly reduced sample-to-sample variations in RUD calculation: Similar biological samples processed at different times had closer normalized RUD (nRUD) values than not normalized ones.

### Dynamic Regulation of 3′UTR by APA in Reprogramming of Different Cell Types

**Figure 2:**
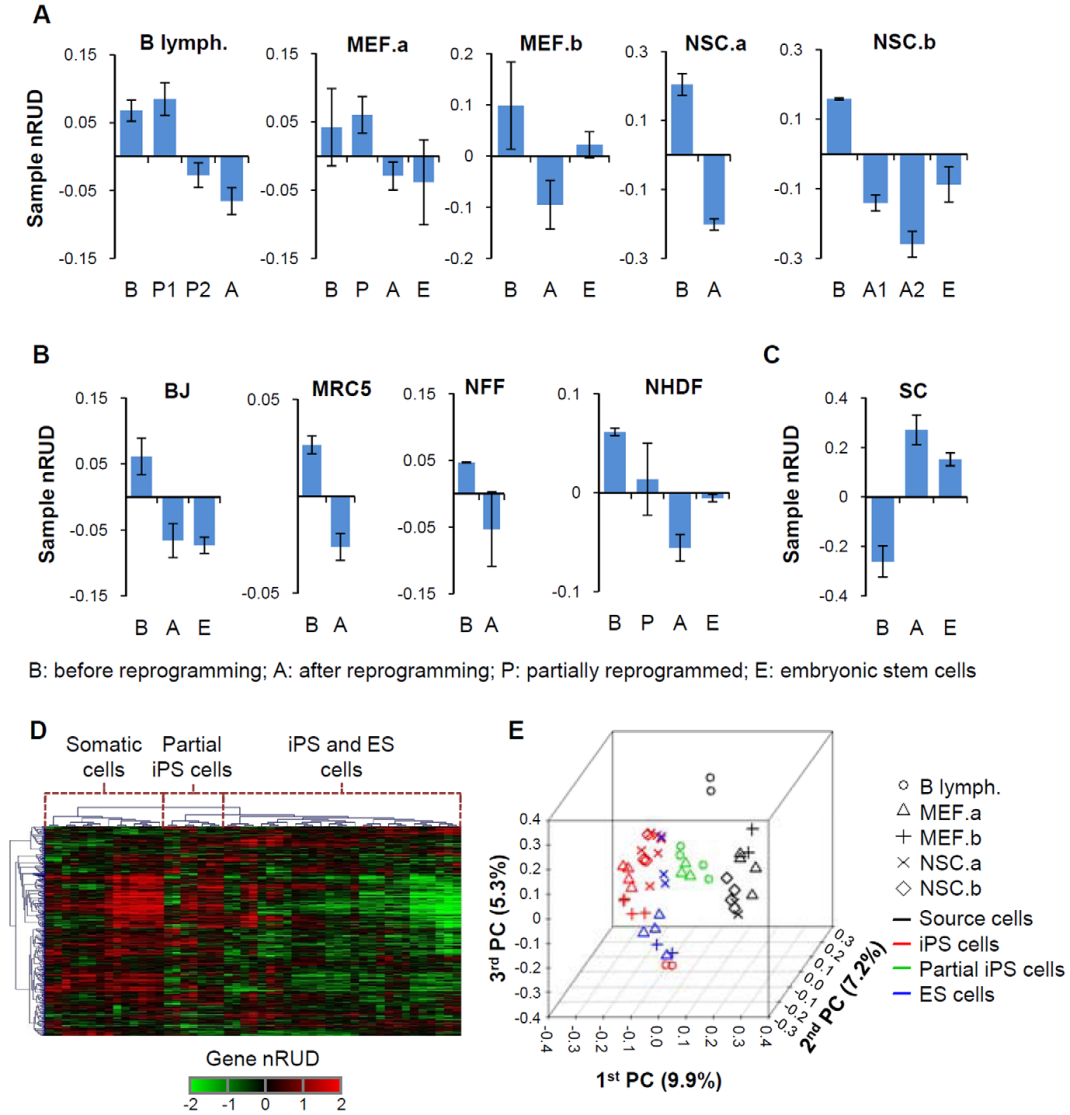
Dynamic regulation of 3′UTR in generation of iPS cells. (A) Mouse cells reprogrammed to iPS cells. Each study is named by its source cell, which is indicated in the graph and listed in Table S1. B lymph., B lymphocyte; MEF, mouse embryonic fibroblast; NSC, neural stem cell. The y-axis for each plot is normalized Relative Usage of Distal poly(A) site score for a sample (nRUD, see Figure S1 for detail). Error bars are standard deviations based on multiple samples. As indicated in the graphs, cells before and after reprogramming are indicated by ‘B’ and ‘A’, respectively, and partially reprogrammed cells and embryonic stem cells (ESCs) are indicated by ‘P’ and ‘E’, respectively. For the B lymph. data set, P1 and P2 corresponds to BIV1 (+Dox) and BIV1 (−Dox) in [32], respectively. (B) Human cells reprogrammed to iPS cells. Data are presented as in (A). All cells were derived from fibroblasts (see Table S1 for details). (C) Generation of iPS cells from human spermatogonial cells (SCs). Data are presented as in (A). (D) Hierarchical clustering of samples and gene nRUD values. Detailed view of the sample cluster and sample names are shown in Figure S7. A total of 674 genes were used. Clustering was based on Pearson Correlation. (E) Principal component (PC) analysis of samples using gene nRUD values. The top 3 PCs are plotted. As shown in the graph, symbols indicate cell types and colors indicate cell states. The percent of variation accounted for by each PC is indicated in parentheses. For all data sets, nRUD values before reprogramming are significantly different than those after reprogramming (*P*<0.05, T-test, see Table S1 for list of *P*-values).

Using nRUD we found that genes tended to express mRNAs with shorter 3′UTRs in iPS cells than in source somatic cells (Figures 2A and 2B). By contrast, 3′UTRs lengthened during generation of iPS cells from SC (Figure 2C). For data sets that included ES cells, the 3′UTR length in reprogrammed iPS cells was generally closer to ES cells than to source cells, which was consistent with their phenotypes and gene expression profiles. The dynamics of 3′UTR regulation in cell reprogramming can be clearly manifested in the NSC.a study, which included both reprogramming of NSCs to iPS cells and differentiation of reprogrammed cells back to NSCs (Figure S4).

Three studies included cells that were partially reprogrammed (B lymph. and MEF.a in Figure 2A and NHDF in Figure 2B), which were believed to be trapped at intermediate stages of reprogramming. Interestingly, the 3′UTR lengths of mRNAs in these partially reprogrammed cells appeared to be longer than those in fully reprogrammed ones. The differences appeared to be consistent with their phenotypes. For example, P1 and P2 in the B lymph. data set represented 2 different partially reprogrammed cells, with P2 being closer to fully reprogrammed iPS cells and ES cells based on the gene expression profie and growth behavior [32]. In line with this, the 3′UTR length for P2 was closer to fully reprogrammed cells and P1 was closer to source cells (Figure 2A). This result suggests that regulation of 3′UTR is a continuous process during generation of iPS cells.

We found that while 3′UTR shortening in reprogramming of somatic cells to iPS cells could be discerned for most human and mouse genes and the consistency across data sets was much higher than random (Figure S5), the extent of shortening, however, appeared to be variable for different cell types (Figures S6). Reprogramming of NSC involved more drastic 3′UTR shortening than other cell types, which is similar in extent, but opposite in direction, to reprogramming of SC (Figures 2 and S6). This result indicates that the direction and extent of 3′UTR regulation in generation of iPS cells reflect the difference between source cells and iPS cells. On this note, it has been reported that mRNAs expressed in neuronal cells tend to have longer 3′UTRs than other cell types, whereas those expressed in testes have shorter ones [26], [28]. In addition, each cell type has a set of genes with a different direction of 3′UTR regulation than the global trend of the cell (Figure S6C), suggesting cell-specific regulation of APA for certain genes. In support of this notion, we found that the APA pattern can be used to separate samples according to reprogramming state and cell type by hierarchical clustering (Figure 2D) and principal component analysis (Figure 2E).

### Regulation of 3′UTR in Generation of iPS Cells Is Related to That in Embryonic Development

**Figure 3:**
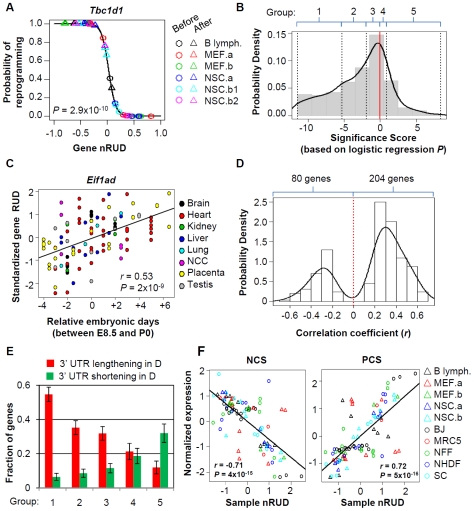
The 3′UTR regulation in generation of iPS cells is related to that in embryonic development. (A) An example of 3′UTR regulation in generation of mouse iPS cells. The gene *Tbc1d1* was randomly selected. The relationship between reprogramming state, i.e. before and after reprogramming, and gene nRUD values from 6 different data sets for 3 different cell types was analyzed by logistic regression. Y-axis is predicted probability that a sample is iPS cell after fitting the logistic regression model. *P*-value for data fitting is shown in the graph. (B) Distribution of logistic regression significance score (SS) for all surveyed genes (674 in total) (See Methods for detail). As indicated on top of the graph, genes with negative SS were evenly divided into 3 groups and those with positive SS were divided into 2 groups. (C) An example of gene (*Eif1ad*) with 3′UTR lengthening in embryonic development. X-axis is relative embryonic days, with 0 being the median time point for each sample set. Y-axis is standardized RUD values which makes different sample sets comparable. Pearson correlation (*r*) and *P*-value for the linear regression line are shown in the graph, which reflect change of 3′UTR length over developmental time. (D) A total of 606 genes with APA that had detectable signals in more than 50% of all samples between 8.5 and P0 were surveyed, and 284 genes had significant change of 3′UTR over time (*P*<0.05). A histogram of correlation (*r*) for these genes is presented. (E) Comparison of 3′UTR regulation in generation of iPS cells and embryonic development. Genes with significant regulation of 3′UTRs by APA in embryonic development were shown in (D). Fractions of genes with lengthening (red) or shortening (green) 3′UTRs during embryonic development for each of the 5 groups derived from (B) are plotted. *P*-value (Chi-squared test comparing fractions of genes with 3′UTR lengthening in embryonic development in 5 groups with those with 3′UTR shortening) = 8.5×10^−11^. (F) Correlation between nRUD values in generation of iPS cells and mRNA expression of the genes that are negatively (left) and positively (right) correlated with 3′UTR length in embryonic development. These gene sets are called negative correlation set (NCS, 59 genes) and positive correlation set (PCS, 74 genes).

Generation of iPS cells involves reprogramming of differentiated cells into an undifferentiated state, analogous to reversal of development. We thus wanted to know how APA in generation of iPS cells was related to that in embryonic development, during which 3′UTRs progressively lengthen [18]. To this end, we focused on mouse genes because of availability of microarray data for mouse embryonic development. For all mouse genes with APA surveyed in this study, we first modeled change of 3′UTR length against 2 reprogramming states, i.e. before and after reprogramming, in 6 cell lines using logistic regression (see Methods for detail) [40]. The *P*-value of the model reflects the correlation between 3′UTR change and reprogramming state. An example is shown in Figure 3A. As expected, there were significantly more genes having negative correlation than those having positive correlation (Figure 3B), consistent with general shortening of 3′UTR during reprogramming of somatic cells.

We next calculated correlation between 3′UTR length and embryonic development stages by Pearson Correlation, using data sets corresponding to 8 different tissues from embryonic day (E) 8.5 to postnatal day (P) 0 [18]. An example is shown in Figure 3C. We then selected genes with significant 3′UTR regulation in embryonic development (*P*<0.05, Figure 3D), and examined how they were regulated in generation of iPS cells. As shown in Figure 3E, genes with 3′UTR shortening in generation of iPS cells were more likely to have 3′UTR lengthening in embryonic development (groups 1–3), whereas genes with 3′UTR lengthening in generation of iPS cells were more likely to have 3′UTR shortening in embryonic development (groups 4 and 5). This result indicates that 3′UTR regulation in generation of iPS cells from somatic cells is largely reversal of that in embryonic development.

We previously selected 2 sets of genes whose mRNA expression levels positively or negatively correlated with 3′UTR length in embryonic development [18]. They were called Positive Correlation Set, (PCS) and Negative Correlation Set (NCS), respectively. Intuitively, they are marker genes for 3′UTR length in cells. We examined how their expression changes correlated with 3′UTR changes in generation of iPS cells, including all source cells, and partially and fully reprogrammed cells. As shown in Figure 3F, mRNA expression changes of both NCS genes and PCS genes significantly (*P* = 4×10^−15^ and 5×10^−16^) correlated with 3′UTR length with good negative and positive *r* values (*r* = −0.71 and 0.72), respectively, further indicating the close relation between generation of iPS cells and embryonic development in 3′UTR regulation.

### Regulation of mRNA Polyadenylation Activity May Be Responsible for 3′UTR Dynamics

**Figure 4:**
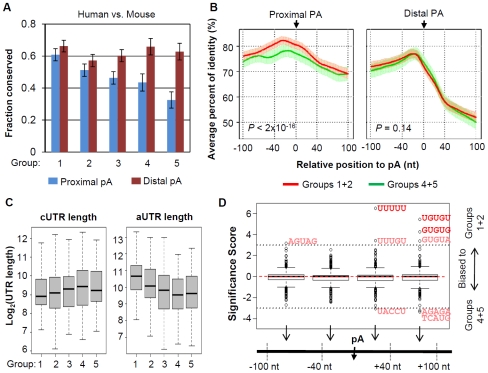
Analysis of poly(A) sites responsible for 3′UTR regulation. (A) Conservation of proximal and distal poly(A) sites between human and mouse genomes for different groups of genes. Gene groups are based on Figure 3B. *P*-value (Chi-squared test with null hypothesis being no difference between groups) = 4.1×10^−4^ for proximal poly(A) sites, and = 0.45 for distal sites. (B) Conservation of sequence surrounding proximal (left) and distal (right) poly(A) sites for groups 1+2 (red line) and groups 4+5 (green line). Y-axis is average percent of identity for a given nucleotide position, which was calculated using genome alignments of human, mouse, rat, and dog. X-axis is relative position to poly(A) site, with the cleavage site set at position 0. Standard errors are indicated by vertical bars along the lines. *P*-values are based on Wilcoxon matched-pairs test comparing 2 conservation profiles from −100 nt to +100 nt. Curves were smoothed by the Lowess regression method. (C) Distribution of cUTR length (left) and aUTR length (right) for genes in the 5 groups. *P*-value (Wilcoxon rank sum test) = 0.096 for cUTR length difference between groups 1+2 and groups 4+5, and = 2.7×10^−9^ for aUTR length difference. (D) Comparison of frequency of occurrence for all 5-mers in different regions surrounding proximal poly(A) sites for genes in groups 1+2 vs. those in groups 4+5. As indicated in the graph, 4 regions were examined, i.e. −100 to −41 nt, −40 to −1 nt, +1 to +40 nt, and +41 to +100 nt. The poly(A) site was set at position 0. Y-axis is the significance score (see Methods for detail). Pentamers with significance score >3 or <−3 are shown in red. Significant ones after *P*-value correction by the Benjamin-Hochberg method are shown in dark red, i.e. UUUUU, UGUGU, and GUGUG.

Alternative 3′UTRs are generated by alternative use of poly(A) sites. Using human and mouse orthologous poly(A) sites [41], we found that the proximal poly(A) sites of genes with more significant 3′UTR shortening in generation of iPS cells tend to be more conserved (Figure 4A). By contrast, there is no difference between distal poly(A) site groups, suggesting that regulation of 3′UTRs by APA is chiefly through proximal poly(A) sites. In addition, using alignments of human, mouse, rat, and dog genomes, we found that sequences surrounding proximal poly(A) sites involved in 3′UTR shortening (groups 1 and 2) were more conserved than those involved in lengthening (groups 4 and 5), whereas those surrounding distal poly(A) sites showed no such differences (Figure 4B).

We found also that genes with more significant 3′UTR shortening in generation of iPS cells had significantly longer aUTRs (Figure 4C), for example *P*-value = 2.7×10^−9^ (Wilcoxon rank sum test) for groups 1+2 vs. groups 4+5. But this trend was not detected for cUTRs. This result may suggest that APA events involving longer aUTRs are more likely to be detected by our method. However, given the trend in conservation of proximal poly(A) sites (Figures 4A and 4B), it is more likely that the proximal sites with longer aUTRs are more easily regulated.

We next reasoned that proximal poly(A) sites that were differentially regulated in generation of iPS cells and embryonic development might be surrounded by different cis elements. To this end, we compared the frequency of occurrence for all 5-mers near proximal poly(A) sites of genes in groups 1 and 2 vs. those in groups 4 and 5. We analyzed 4 regions, i.e. −100 to −41 nt, −40 to −1 nt, +1 to +40 nt, and +41 to +100 nt surrounding the poly(A) site. As shown in Figure 4D, we found that the major differences between these groups were elements located downstream of poly(A) sites, including UUUUU, UGUGU, and GUGUG, which were the binding sites for CstF-64, a factor in the CstF complex of the polyadenylation machinery [42], [43], suggesting that the activity of CstF complex may be regulated during generation of iPS cells.

**Figure 5:**
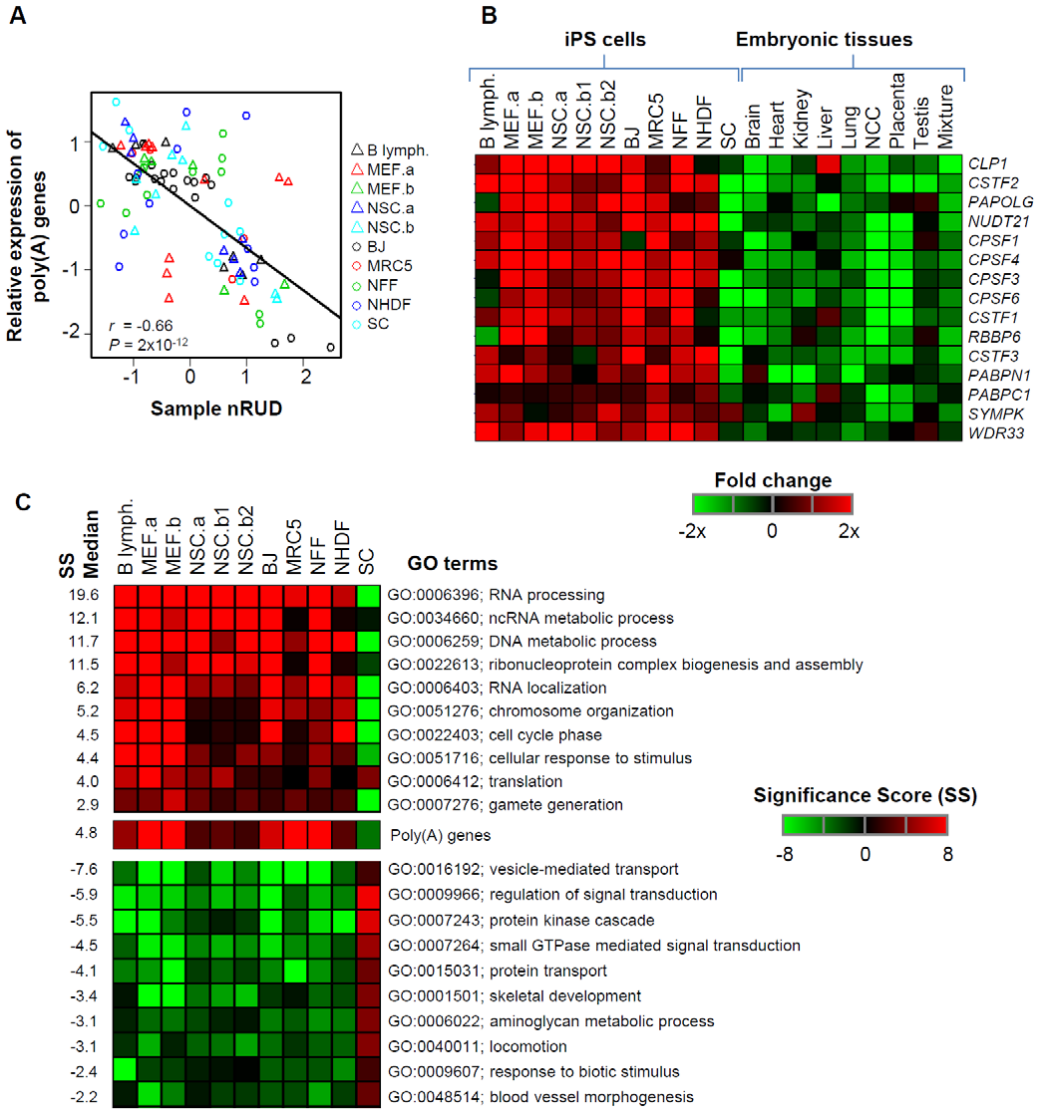
Gene expression analysis in generation of iPS cells. (A) Correlation between mRNA expression of 94 poly(A) genes and nRUD values for all cell types, including before and after reprogramming and partially reprogrammed cells. Poly(A) genes are those reported in [21] plus gene encoding Clp1 (see Table S3 for the complete list). (B) A list of genes encoding core polyadenylation factors that are consistently regulated in generation of iPS cells. Fold changes (ratio of after reprogramming to before reprogramming) are shown in a heatmap according to the color scale shown at the bottom. Only the genes with consistent trend of regulation, either upregulation or downregulation in >9 out of 10 data sets, during reprogramming of somatic cells are shown. Data for SC and different tissues in embryonic development are also shown for comparison. Human gene symbols are used to annotate genes. (C) Gene Ontology (GO) terms that are significantly associated with genes upregulated (top) and downregulated (bottom) during generation of human and mouse iPS cells from somatic cells. Significance score (SS) is used to represent *P*-values (see Methods for detail), and is shown in a heatmap according to the scale shown in the graph. The poly(A) gene group was also analyzed and is shown in the middle. Its *P*-values are <0.01 for all cell types. The median SS based on reprogramming of somatic cells is listed and used to sort GO terms. GO terms associated with more than 1,500 genes are considered too generic and are discarded. To eliminate redundancy, we require that the reported GO terms do not overlap with any other GO term with greater SS by more than 25% of associated genes.

We then analyzed expression of 94 genes encoding proteins that are part of or associate with the mRNA polyadenylation machinery, largely based on a recent proteomics study [21]. For simplicity, they were called poly(A) genes (see Table S3 for the complete list). Overall, these poly(A) genes were significantly upregulated during somatic cell reprogramming (Figure S8) and there existed a significant negative correlation between expression of these genes and 3′UTR length (Figure 5A) across all data sets including SC and partially reprogrammed cells, suggesting that regulation of polyadenylation activity may be responsible for the regulation of 3′UTR in cell reprogramming. For example, 15 out of 23 genes encoding core polyadenylation factors (Figure 5B) and 31 out of 71 genes encoding associated factors (Figure S9) were consistently upregulated during generation of iPS cells across data sets, most of which were downregulated during reprogramming of SC and embryonic development. Interestingly, all 3 genes encoding factors in the CstF complex were significantly regulated, which was in good agreement with the cis element result described above. In addition, most of the genes encoding the CPSF complex were also upregulated, suggesting coordination in gene expression between CPSF and CstF complexes.

To understand the significance of regulation of poly(A) genes in a global context, we analyzed all Gene Ontology (GO) Biological Processes (BPs) with respect to regulation of associated genes in generation of iPS cells. As shown in Figure 5C, consistent with 3′UTR regulation, ‘RNA processing’, which included over half of the 94 poly(A) genes, was the most significantly upregulated BP in reprogramming of somatic cells. Significantly, poly(A) genes, if treated as a BP group, would rank 7^th^ based on *P*-value in upregulated BPs. In addition, consistent with our previous finding that 3′UTR regulation coincides with regulation of genes involved in proliferation, differentiation, and morphogenesis during embryonic development, many related BPs were significantly regulated, most of which in opposite directions in generation of iPS cells from somatic cells compared with embryonic development. Interestingly, similar to 3′UTR regulation, most of the significant BPs for generation of iPS cells from somatic cells were also significant in reprogramming of SC, but in opposite directions, indicating substantial differences between germ cells and somatic cells in gene expression during the reprogramming process.

### Impact of 3′UTR Regulation on Post-Transcriptional Gene Regulation in Generation of iPS Cells

**Figure 6:**
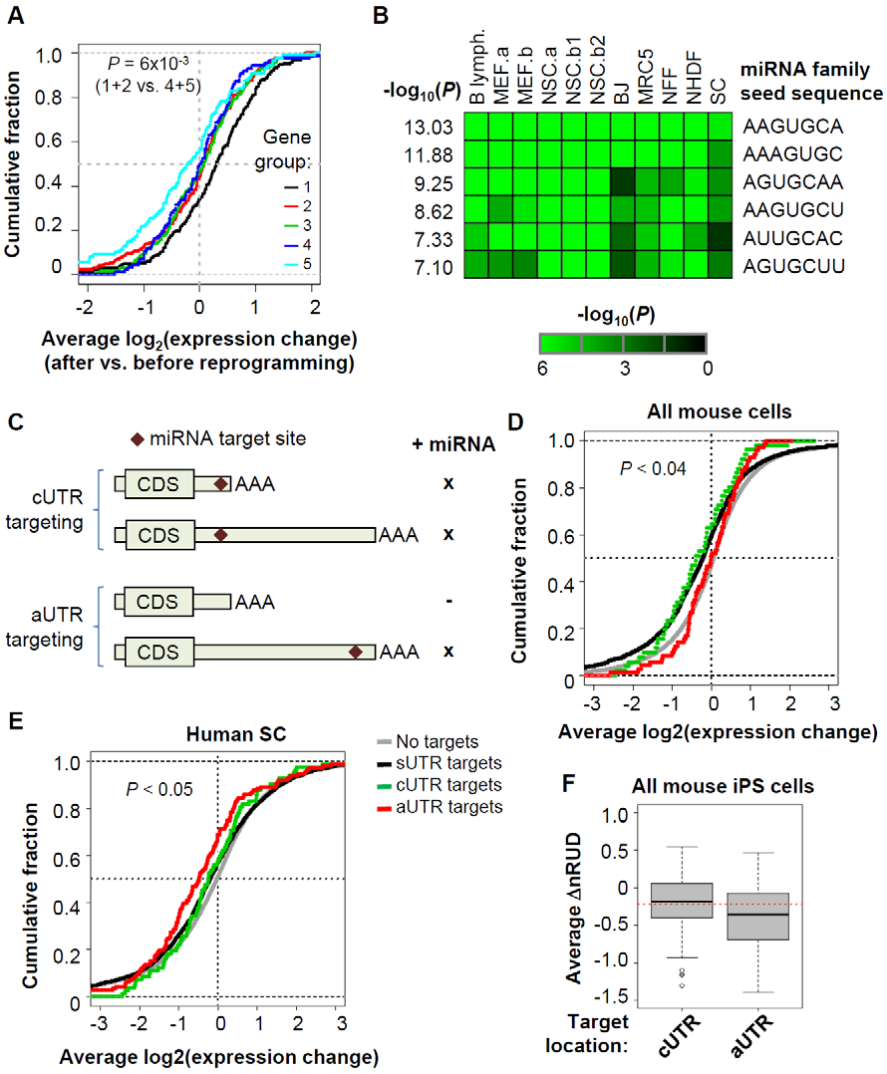
Impact of 3′UTR dynamics on gene expression and miRNA targeting. (A) Distribution of expression changes for genes with different 3′UTR regulations. Gene groups are based on Figure 3B. For each gene, the ratio of cUTR probe intensity after reprogramming to that before reprogramming was calculated and averaged across data sets. The Kolmogorov-Smironov test was used to compare the combined distribution of groups 1+2 with that of groups 4+5. (B) miRNA families predicted to function in iPS cells. The *P*-values (Fisher's exact test) for significance of downregulation of miRNA target are shown in a heatmap based on the color scale shown in the graph. Seed sequences are shown to represent the miRNA families. AAGUGCA is the seed sequence for miR-291b-3p/519a/519b-3p/519c-3p, AAAGUGC for miR-17-5p/20/93.mr/106/519.d, AGUGCAA to miR-130/301, AAGUGCU for miR-106/302, AUUGCAC for miR-25/32/92/92ab/363/367, and AGUGCUU for miR-302ac/520f. (C) A model showing the impact of 3′ UTR dynamics on miRNA targeting. “x” indicates miRNA targeting, and ‘−’ indicates no miRNA effect. (D) Cumulative fraction of change of expression for different groups of genes based on miRNA target site location. Only the conserved target sites for miRNA families shown in (B) are used. The change of expression is based on ratio of after reprogramming to before reprogramming for probes targeting cUTRs, and is average of 5 mouse data sets, i.e. B lymph., MEF.a, MEF.b, NSC.a, NSC.b1, and NSC.b2. Different groups are colored differently as indicated in the graph. (E) As in (D), only the result using human SC data is presented. (F) Average ΔnRUD for genes with miRNA target sites in different UTR regions. Only the genes with target sites for the 6 miRNA families shown in (B) are used. Red dotted line indicates average ΔnRUD for all surveyed genes.

Regulation of 3′UTR length by APA can impact on cis elements located in 3′UTRs, resulting in different mRNA metabolism for different mRNA isoforms. Generally, cis elements in the 3′UTRs function to inhibit gene expression by repressing translation or facilitating mRNA degradation, such as miRNA target sites and AU-rich and GU-rich elements. Consistent with this notion, we found that genes with significant 3′UTR shortening in generation of iPS cells from somatic cells (group 1) were significantly less downregulated than other genes (Figure 6A).

Several miRNAs have been reported to be expressed in ES cells [44], [45] and introduction of ES cell-specific miRNAs was shown to promote generation of iPS cells [46]. To examine how miRNAs were expressed and functioned in iPS cells, we first predicted functional miRNAs using mRNA profiles of their target genes. This analysis was based on the rationale that mRNAs targeted by miRNAs would show downregulated expression profiles compared with non-target mRNAs using microarrays [47], [48]. We focused on 211 miRNA families that were conserved between human and mouse (Table S4), and used the TargetScan method to predict target sites [13] with requirement of conservation in human, mouse, rat, and dog (see Methods for detail). In good agreement with previous reports [49], [50], we found that miRNA target sites tended to be located near 5′ and 3′ ends for all UTR groups (Figure S10), i.e. sUTR, cUTR, and aUTR. Using Fisher's exact test, we calculated significance score for each miRNA family which indicated whether its target genes were significantly upregulated or downregulated compared with other genes. By this method, we predicted a number of miRNA families which were likely to be functional in each iPS cell line (see Table S4 for the full list). Significantly, the top 6 miRNAs with consistently high significance scores across all data sets were all reported to be expressed in ES cells (Figure 6B)[44], [45], indicating concordance between iPS cells and ES cells with respect to miRNA-mediated gene regulation.

We next reasoned that genes with miRNA target sites located in aUTRs might be regulated differently than genes with sites located in cUTRs because of change of 3′UTR length during generation of iPS cells [51], as illustrated in Figure 6C. We focused on target sites for the top 6 miRNA families that were reported to be expressed in ES cells. As shown in Figure 6D, genes with target sites in aUTRs were significantly (*P*<0.04) less downregulated than those with target sites in cUTRs or sUTRs during generation of mouse iPS cells from somatic cells, suggesting that shortening of 3′UTRs leads to evasion of miRNA targeting in aUTRs. In line with this finding, genes with aUTR targets tended to be more downregulated than those with target sites in cUTRs or sUTRs during reprogramming of human SC, during which 3′UTRs were lengthened (Figure 6E). Furthermore, we also found that change of 3′UTR length during reprogramming was more significant for genes with miRNA target sites in aUTRs than those with sites in cUTRs (Figure 6F), suggesting that mRNA isoforms with aUTRs were selectively degraded, leading to relatively higher abundance of isoforms with only cUTRs.

## Discussion

Here we show that 3′UTRs are reprogrammed by APA during generation of iPS cells from different cell types, which appears to be reversal of their regulation in development: Shortening of 3′UTRs in reprogramming of somatic cells is opposite to their regulation in embryonic development, and lengthening of 3′UTRs in reprogramming of germ cells appears to be reversal of their regulation in postnatal development of testis [18]. These results underline the dynamic nature of 3′UTR regulation in development, and indicate that APA is an integral part of cell reprogramming process. Interestingly, the developmental potency of a cell type, i.e. germ cells>ES cells>partially differentiated cells > terminally differentiated cells, seems to inversely correlate with the global 3′UTR length. On this note, it remains to be seen whether the 3′UTR length can be used as a marker to monitor cell reprogramming process, and whether perturbation of APA may alter the efficiency of generation of iPS cells.

**Figure 7:**
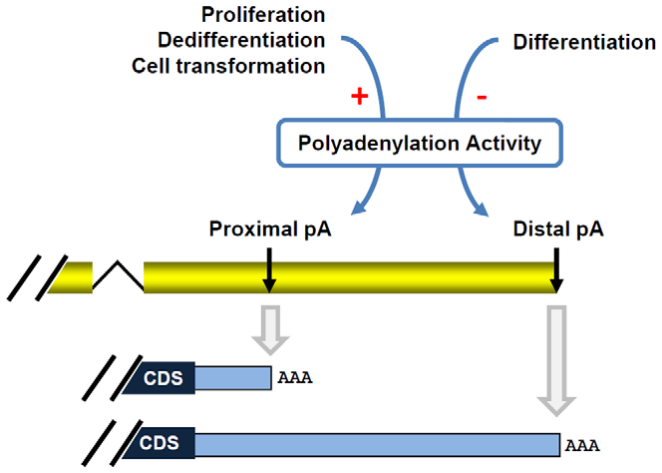
A model for regulation of 3′UTR by APA in proliferation/differentiation. During proliferation, dedifferentiation, and cell transformation, high mRNA polyadenylation activity leads to usage of proximal poly(A) sites, whereas during differentiation, low mRNA polyadenylation activity leads to usage of distal poly(A) sites. The signs ‘+’ and ‘−’ indicate activation and inhibition, respectively.

Sandberg et al. reported 3′UTR shortening in T cell activation and a general correlation between 3′UTR shortening and cell proliferation [30]. Mayr and Bartel further found that 3′UTR shortening is more apparent in transformed cells than nontransformed ones with similar proliferating rate [31]. Taken together their findings and ours, we propose that the 3′UTR lengthen is controlled by both proliferation and differentiation states, as depicted in Figure 7. Given the good correlation between expression of poly(A) genes and global 3′UTR length, one attractive model for the underlying mechanism is that the polyadenylation machinery is dynamically regulated during proliferation/differentiation. In line with this, we found that binding sites of several transcription factors related to proliferation/differentiation, including E2F, c-myc and p53, are enriched in the promoter regions of RNA processing genes (Figure S11), which included more than half of the poly(A) genes, and factors in E2F and pRB families were reported to bind promoter regions of genes encoding CstF factors [52].

By comparing 3′UTR dynamics in cell reprogramming with that in embryonic development, we uncovered several features that are important for regulation of proximal poly(A) sites: 1) The proximal sites that are highly responsive to change of cell state during cell reprogramming, such as group 1 poly(A) sites in Figure 3B, are also highly regulated in embryonic development, leading to 3′UTR shortening and lengthening in reprogramming and development, respectively. 2) Compared with other sites, these highly regulatable ones are more conserved across species, indicating their functional importance. Their flanking regions are under higher evolutionary constraint, suggesting more elements may be located around these sites, which may function under different conditions. On this note, it was recently reported that the proximal poly(A) sites having higher variation of usage in different human tissues tend be to flanked by sequences with higher conservation rate [27]. 3) Highly regulatable proximal poly(A) sites lead to longer aUTRs when not used. On the one hand, this suggests that more cis elements in aUTRs can be regulated by APA for the corresponding genes; on the other hand, longer aUTRs may allow more effective regulation of proximal poly(A) sites with less interference from distal poly(A) sites. 4) Highly regulatable proximal sites have higher occurrences of downstream U-rich and GU-rich elements, which are the binding sites for the CstF complex. While the roles of elements located upstream or beyond our investigated region (−100 to +100 nt) cannot be ruled out, this result argues that ability to recruit the CstF complex is crucial for regulatability of a proximal poly(A) site when cellular polyadenylation activity changes during proliferation/differentiation. Presumably, this is due to the fact that proximal poly(A) sites have weaker upstream PAS compared with distal poly(A) sites [16], and thus are more susceptible to regulation by downstream elements. This finding is also consistent with an early discovery that CstF-64 regulates APA of IgM heavy chain gene in B cell differentiation [53].

Interestingly, by GO analysis we found that genes in group 1 tend to have functions in signal transduction, such as protein ubiquitination and phosphorylation, RNA processing, and cell cycle process (Table S5). As shown previously and here, 3′UTR lengthening and shortening can impact on miRNA targeting, mRNA stability, and protein production. Since these genes are highly regulated in cell proliferation and differentiation and may play roles in APA, it is possible that regulation of their 3′UTRs by APA forms feedforward and feedback regulatory circuitries, which can ensure rapid regulation of their gene expression during cell proliferation and differentiation. This is to be examined in detail in the future.

While the general trend of 3′UTR regulation is consistent across different somatic cell types, the extent of regulation varies. Noise in analyzing heterogeneous data sets can be a contributing factor. However, the good sample clustering (Figures 2D and 2E) according to cell type and state using gene nRUD argues against that it is a major one. The difference in general polyadenylation activity can explain some of the variation, given the correlation between expression of poly(A) genes and sample nRUD (Figure 5A). However, cell-specific regulation is very likely to contribute additional variations in 3′UTR regulation. For example, NSCs have the most drastic shortening of 3′UTRs of all cell types analyzed, but poly(A) genes do not appear to be more regulated than in other cells (Figure 5C). Thus, other cell-specific APA regulatory mechanisms are in play in addition to regulation of general polyadenylation factors. In support of this notion, a growing number of factors have been shown to regulate polyadenylation [24]. For example, the RNA binding protein Nova has been shown to have a widespread impact on APA in neuronal cells [54]. Conversely, since the APA pattern is determined by both cell type and developmental state, it can be used as biomarker for sample clustering and classification. Since the APA pattern is calculated by comparing different isoforms, the data are internally normalized, and can be more robust than mRNA levels for separating samples.

Our RUD values were based on microarray probes targeting cUTR and aUTRs that are defined by proximal and distal poly(A) sites. Notably, about 40–50% human and mouse genes with APA contain also poly(A) sites between proximal and distal sites, which we previously named ‘middle’ poly(A) sites [16]. Analysis of middle poly(A) sites would require dividing aUTR probes into different groups, i.e. upstream and downstream of each middle poly(A) site. Due to the limited number of microarray probes for aUTRs, only a very small number of genes can be examined by this approach. Therefore, while we did observe higher probe-to-probe variations within aUTRs than those within cUTRs (data not shown), which may be partially attributable to usage of middle poly(A) sites, we grouped all aUTR probes together in this study. In a sense, our analysis detects only a general trend of upstream poly(A) site usage vs. downstream site usage. Since middle poly(A) sites are usually less frequently used than proximal and distal sites [16], [41], variation of their usage would have only a marginal effect, if any, on median-based sample RUD and detection of global trend of 3′UTR lengthening or shortening. On the other hand, it is noteworthy that regulation of some middle poly(A) sites can be functionally relevant, as a sizable fraction of these sites are conserved in mammals, and some tissues appear to favor their usage [26]. The technical limitation in analysis of middle poly(A) sites can be significantly mitigated when genome-tiling microarrays or deep sequencing techniques are employed, which are just beginning to be used in iPS cell research.

We found that microarray data generated at different times for the same biological sample can give different 3′UTR length measurements, suggesting systematic variation in sample processing. One possibility is difference in reverse transcription which starts at the 3′ end and stops at regions that are variable between samples. Presumably, this can not only affect analysis of 3′ UTRs but also gene expression. Thus, precautions need to be taken when data sets from different studies or sample processing times are compared. In this study, we used sUTRs to normalize RUD values derived from cUTRs and aUTRs. While it is possible that some sUTRs may contain alternative poly(A) sites that are yet to be identified by the PolyA_DB database used in this study [55], the method appeared to significantly reduce sample-to-sample variations. In theory, this approach can also be applied to other platforms, such as genome-tiling microarray and deep sequencing.

## Methods

### Microarray Data Analysis

Microarray data were downloaded from the NCBI GEO database and are listed in Table S1. The data set for embryonic development of 8 tissues were described in [18]. Gene expression analysis was carried out by the Affymetrix Expression Console software using the Robust Multi-array Average (RMA) method for normalization. The MAS 5.0 program was used to get ‘absent’ and ‘present’ calls. Affymetrix GeneChip probes were mapped to cUTR and aUTR sequences as previously described [18]. Poly(A) sites in human and mouse genomes were obtained from PolyA_DB [55]. To ensure data quality, we used only those aUTRs supported by RefSeq, and required at least 2 probes in both cUTR and aUTR for each gene. The numbers of probes used for cUTRs and aUTRs are shown in Figure S2. Analysis of 3′UTR regulation was based on ratios of cUTR probe intensities to aUTR ones across a sample set, as illustrated in Figure S1. Each gene was assigned a value named Relative Usage of Distal poly(A) site score, or RUD, reflecting relative 3′UTR length in a given sample. The median RUD of all genes in a sample is RUD for the sample.

We found that in some cases, RUD can be influenced by experimental design. As shown in Figure S3, a batch of samples processed together could have different RUD values than another batch which was not consistent with biological grouping. This systematic variation might be due to technical differences between batches of microarray experiments, such as reverse transcription, labeling, hybridization, etc. To control this variation, we included a normalization step using genes without APA, as described in detail in Figure S1. The normalized RUD value is called nRUD.

### Analysis of APA Regulation

A logistic regression model was employed to examine the correlation between nRUD values and reprogramming states, i.e. iPS or somatic cells. Let *p* be the probability that a sample is iPS cell. We define *logit* (*p*) = *ln* (*p*/1−*p*) = *a*+b*x, where *x* is a vector of nRUD values; *a* and *b* are coefficients to be estimated. Chi-square test was used to derive a *P*-value for the model fitting. The significance of APA regulation during reprogramming was represented by a significance score (SS). SS = log_10_ (*P*-value)*s, where s = 1 when b<0, and s = −1 otherwise. Thus, a negative SS indicates 3′UTR shortening in cell reprogramming, and a positive value for lengthening.

### Poly(A) Site Analysis

Conservation of poly(A) sites was analyzed by the UCSC liftover program using human and mouse genome alignments, allowing +/−24 nucleotides (nt) for finding orthologous sites, as described in [41]. Sequence conservation of the poly(A) region (−100 to +100 nt) was based on the genome alignment of human, mouse, rat and dog, obtained from UCSC MultiZ files. The average percent of identity value at each position was calculated to indicate the conservation rate for the position. To identify potential cis elements associated with 3′UTR regulation by APA, occurrences of all 5-mers in the poly(A) regions were enumerated. Poly(A) sites were divided into 5 groups based on regulation of 3′UTR in generation of iPS cells (Figure 3B). The Fisher's exact test was used to examine the significance of association between a 5-mer and gene groups, resulting in *P*-value 1 for bias to groups 1+2 and *P*-value 2 for bias to groups 4+5. A significance score (SS) was used to represent the overall significance. SS = −log_10_ (*P*-value 1) when *P*-value 1<*P*-value 2, and = log_10_(*P*-value 2) otherwise.

### Gene Ontology (GO) Analysis

We used NCBI GO annotations for genes. Only the Biological Process (BP) category was used. The GO Parser program from BioPerl was used to find all associated GO terms for a given gene. Only genes with detectable signals based on A/P call of MAS 5.0 in >50% of samples were used. Fold change >1.2 and *P*-value <0.05 (t-test) between before and after reprogramming samples were used to select regulated genes. We used the Fisher's exact test to examine whether a significant fraction of genes associated with a GO term were upregulated (*P*-value 1) or downregulated (*P*-value 2). Each GO term was given a significance score (SS) based on the *P*-values. SS = −log_10_ (*P*-value 1) when *P*-value 1<*P*-value 2, and = log_10_(*P*-value 2) otherwise.

### miRNA Analysis

We used miRNA seed matches to identify miRNA target sites by the TargetScan 4.1 program [13]. Target sites were those with matches at seed region (2–7 nucleotides, nt) and either M8 or A1. We required the seed region to be conserved among human, mouse, rat, and dog genomes. The alignments of UTR sequences were obtained from UCSC. miRNA information was obtained from the TargetScan 5.1 web download page. miRNAs were grouped into families based on the 2–8 nt region and only those conserved between human and mouse in the seed region were used (211 families in total). To predict miRNA effects, we used Fisher's exact test to examine whether a significant fraction of targeted genes for a given miRNA were upregulated (*P*-value 1) or downregulated (*P*-value 2) using fold change >1.2 and *P*<0.05 (t-test) as cutoff. Significance score (SS) was then derived as described above for SS in GO analysis.

### Promoter Analysis

Position-specific scoring matrices (PSSMs) for transcription factor binding sites (TFBS) were obtained from the Transfac database (Version 11.4). Only those with quality score 1–4 were used. The transcription start sites (TSS) were defined by RefSeq. The MATCH tool [56] was used to scan the −700 nt to +300 nt promoter region surrounding TSS with the mode to minimize false negative. We required all hits to be 100% conserved between human and mouse genomes. Fisher exact test was used to identify TFBS that were significantly associated with RNA processing genes. For factors with multiple PSSMs, the one with the most significant *P*-value was shown.

## Supporting Information

Figure S1 Analysis of APA using normalized Relative Usage of Distal poly(A) site score (nRUD). Method to calculate nRUD. Top, a gene with APA; Bottom, a gene without APA. CDS, coding sequence; cUTR, constitutive UTR; aUTR, alternative UTR; sUTR, single UTR; AAA, poly(A) tail. Red bars are Affymetrix (Affy) GeneChip probes.
(0.02 MB PDF)

Figure S2 Histograms of cUTR and aUTR probe numbers for Affymetrix HU133 v2.0 GeneChip and Mouse 430 v2.0 GeneChip. All human data sets used Hu133 v2.0 and all mouse data sets used Mouse 430 v2.0.
(0.01 MB PDF)

Figure S3 Correction of systematic differences between data sets using the nRUD method. (A) Data set for the generation of iPS cells from human BJ fibroblast (BJ in Figure 1). (B) RUD values without normalization. (C) nRUD values, i.e. RUD values with normalization. Samples in the same iPS cell group have more consistent nRUD values than original RUD values. (D) RUD' values derived from probes targeting sUTRs. The difference between February/March samples and May samples indicates systematic differences in sample processing.
(0.02 MB PDF)

Figure S4 Dynamic regulation of 3′UTR by APA in generation and differentiation of iPS cells. Shortening of 3′UTRs in generation of iPS cells from adult mouse neural stem cells (NSC), and lengthening of 3′UTRs in differentiation of iPS cells to NSC.
(0.02 MB PDF)

Figure S5 Consistent regulation of 3′UTR in generation of iPS cells across different sample sets. (A) Heatmap showing 674 mouse genes with APA surveyed in 6 sample sets. The 3′UTR regulation was measured by gene nRUD, which is represented by color according to the scale shown in the graph, with red indicating 3′UTR lengthening and green 3′UTR shortening. Samples and genes were also clustered using gene nRUD by hierarchical clustering using Pearson Correlation. (B) Genes in (A) with 3′UTR shortening in 5 out of 6 sample sets were selected (47% of total). (C) The percent of selected genes (47%) is significantly higher than expected. The histogram shows distribution of percent of genes having 3′UTR shortening in 5 out of 6 sample sets when genes are randomized in each column. The red line in the graph indicates the observed percent of genes. (D) As in (A), 996 human genes were surveyed. (E) Genes in (D) with 3′UTR shortening in 3 out 4 sample sets were selected, excluding SC. The data for SC are included for comparison. (F) As in (C), the percent of genes selected (49%) is significantly higher than that using randomized data.
(0.28 MB PDF)

Figure S6 Regulation of 3′UTR in generation of iPS cells. (A) Left, distribution of nRUD for genes surveyed in NSC.a; Right, scatter plot of genes with APA. Each dot is a gene surveyed by microarray probes. X-axis and Y-axis are log2(A/B) values for probes targeting cUTRs and aUTRs, respectively, where A and B are average probe intensities for samples after and before reprogramming, respectively. Genes with nRUD greater than 1.5*(standard deviation of all genes) are shown in red, indicating significant 3′UTR lengthening, or green, indicating significant 3′UTR shortening. The numbers of genes for these 2 groups are indicated in insets. (B) Left, distribution nRUD for genes surveyed in SC; Right, scatter plot of genes with APA, as described for (A). (C) Selection of genes with 3′UTR regulation in generation of iPS cells using 3 cutoffs, i.e. 1, 1.5, and 2 standard deviation, as illustrated in (A) and (B). L and S are number of genes with 3′UTR lengthening and shortening, respectively. P-values are based on binomial tests for comparing L and S. (D) S/L and L/S are ratios. Three cutoffs were used to select genes.
(0.04 MB PDF)

Figure S7 Separation of samples using gene nRUD. Sample cluster shown in Figure 2D with sample names.
(0.03 MB PDF)

Figure S8 Poly(A) genes are significantly upregulated during cell reprogramming from different mouse cell types. For each data set, the ratios of gene expression after reprogramming to that before reprogramming for all genes and poly(A) genes were plotted in a cumulative distribution function (CDF) plot (left) and a boxplot (right). The difference between all genes and poly(A) genes is significant (P-value <0.005) for all data sets, based on Kolmogorov-Smironov test and Wilcoxon rank sum test.
(0.05 MB PDF)

Figure S9 Regulation of genes encoding auxiliary polyadenylation factors in generation of iPS cells and embryonic development. For generation of iPS cells, samples before and after reprogramming were compared. Positive values indicate upregulation after reprogramming. For embryonic development, the gene expression values in the first and last days of embryonic development were compared. Samples for 8 individual tissues and mixed tissue were used, as described for Figure 5. Only the genes with consistent trend of regulation, either upregulation or downregulation in 9 out of 10 data sets for somatic cell reprogramming are shown.
(0.02 MB PDF)

Figure S10 Distribution of miRNA target sites in different UTR groups. (A) Distribution of target sites for all 211 conserved miRNA families that are surveyed in this study. X-axis in each graph is relative location of target sites based on 10 evenly divided sub-regions in any given UTR. (B) Distribution of target sites for 6 miRNAs analyzed in this study.
(0.01 MB PDF)

Figure S11 Transcription Factor Binding Sites (TFBS) significantly associated with RNA processing genes. P-values were based on Fisher's exact test. Sequence logos for TFBS are also shown. See Methods for detail.
(0.03 MB PDF)

Table S1 Data sets used in this study.
(0.06 MB PDF)

Table S2 Microarray samples used in this study.
(0.02 MB PDF)

Table S3 Poly(A) genes analyzed in this study.
(0.11 MB PDF)

Table S4 Prediction of function in generation of iPS cells for 211 miRNA families.
(0.05 MB PDF)

Table S5 Significant Gene Ontology terms associated with genes in group 1.
(0.01 MB PDF)

## References

[1] Rossant J (2007). Stem cells and lineage development in the mammalian blastocyst.. Reprod Fertil Dev.

[2] Takahashi K, Yamanaka S (2006). Induction of pluripotent stem cells from mouse embryonic and adult fibroblast cultures by defined factors.. Cell.

[3] Yu J, Thomson JA (2008). Pluripotent stem cell lines.. Genes & Development.

[4] Lowry WE, Plath K (2008). The many ways to make an iPS cell.. Nat Biotechnol.

[5] Zhou H, Wu S, Joo JY, Zhu S, Han DW (2009). Generation of induced pluripotent stem cells using recombinant proteins.. Cell Stem Cell.

[6] Matsui Y, Zsebo K, Hogan BL (1992). Derivation of pluripotential embryonic stem cells from murine primordial germ cells in culture.. Cell.

[7] Kanatsu-Shinohara M, Inoue K, Lee J, Yoshimoto M, Ogonuki N (2004). Generation of pluripotent stem cells from neonatal mouse testis.. Cell.

[8] Guan K, Nayernia K, Maier LS, Wagner S, Dressel R (2006). Pluripotency of spermatogonial stem cells from adult mouse testis.. Nature.

[9] Conrad S, Renninger M, Hennenlotter J, Wiesner T, Just L (2008). Generation of pluripotent stem cells from adult human testis.. Nature.

[10] Wickens M, Anderson P, Jackson RJ (1997). Life and death in the cytoplasm: messages from the 3′ end.. Curr Opin Genet Dev.

[11] Keene JD (2007). RNA regulons: coordination of post-transcriptional events.. Nat Rev Genet.

[12] Garneau NL, Wilusz J, Wilusz CJ (2007). The highways and byways of mRNA decay.. Nat Rev Mol Cell Biol.

[13] Lewis BP, Burge CB, Bartel DP (2005). Conserved seed pairing, often flanked by adenosines, indicates that thousands of human genes are microRNA targets.. Cell.

[14] Bakheet T, Williams BR, Khabar KS (2006). ARED 3.0: the large and diverse AU-rich transcriptome.. Nucleic Acids Res.

[15] Vlasova IA, Tahoe NM, Fan D, Larsson O, Rattenbacher B (2008). Conserved GU-rich elements mediate mRNA decay by binding to CUG-binding protein 1.. Mol Cell.

[16] Tian B, Hu J, Zhang H, Lutz CS (2005). A large-scale analysis of mRNA polyadenylation of human and mouse genes.. Nucleic Acids Res.

[17] Yan J, Marr TG (2005). Computational analysis of 3′-ends of ESTs shows four classes of alternative polyadenylation in human, mouse, and rat.. Genome Res.

[18] Ji Z, Lee JY, Pan Z, Jiang B, Tian B (2009). Progressive lengthening of 3′ untranslated regions of mRNAs by alternative polyadenylation during mouse embryonic development.. Proc Natl Acad Sci U S A.

[19] Colgan DF, Manley JL (1997). Mechanism and regulation of mRNA polyadenylation.. Genes Dev.

[20] Zhao J, Hyman L, Moore C (1999). Formation of mRNA 3′ ends in eukaryotes: mechanism, regulation, and interrelationships with other steps in mRNA synthesis.. Microbiol Mol Biol Rev.

[21] Shi Y, Di Giammartino DC, Taylor D, Sarkeshik A, Rice WJ (2009). Molecular architecture of the human pre-mRNA 3′ processing complex.. Mol Cell.

[22] Hu J, Lutz CS, Wilusz J, Tian B (2005). Bioinformatic identification of candidate cis-regulatory elements involved in human mRNA polyadenylation.. RNA.

[23] Danckwardt S, Hentze MW, Kulozik AE (2008). 3′ end mRNA processing: molecular mechanisms and implications for health and disease.. Embo J.

[24] Lutz CS (2008). Alternative polyadenylation: a twist on mRNA 3′ end formation.. ACS Chem Biol.

[25] Edwalds-Gilbert G, Veraldi KL, Milcarek C (1997). Alternative poly(A) site selection in complex transcription units: means to an end?. Nucleic Acids Res.

[26] Zhang H, Lee JY, Tian B (2005). Biased alternative polyadenylation in human tissues.. Genome Biol.

[27] Wang ET, Sandberg R, Luo S, Khrebtukova I, Zhang L (2008). Alternative isoform regulation in human tissue transcriptomes.. Nature.

[28] Liu D, Brockman JM, Dass B, Hutchins LN, Singh P (2007). Systematic variation in mRNA 3′-processing signals during mouse spermatogenesis.. Nucleic Acids Res.

[29] Flavell SW, Kim TK, Gray JM, Harmin DA, Hemberg M (2008). Genome-wide analysis of MEF2 transcriptional program reveals synaptic target genes and neuronal activity-dependent polyadenylation site selection.. Neuron.

[30] Sandberg R, Neilson JR, Sarma A, Sharp PA, Burge CB (2008). Proliferating cells express mRNAs with shortened 3′ untranslated regions and fewer microRNA target sites.. Science.

[31] Mayr C, Bartel DP (2009). Widespread Shortening of 3′UTRs by Alternative Cleavage and Polyadenylation Activates Oncogenes in Cancer Cells.. Cell.

[32] Mikkelsen TS, Hanna J, Zhang X, Ku M, Wernig M (2008). Dissecting direct reprogramming through integrative genomic analysis.. Nature.

[33] Sridharan R, Tchieu J, Mason MJ, Yachechko R, Kuoy E (2009). Role of the murine reprogramming factors in the induction of pluripotency.. Cell.

[34] Kim JB, Zaehres H, Wu G, Gentile L, Ko K (2008). Pluripotent stem cells induced from adult neural stem cells by reprogramming with two factors.. Nature.

[35] Kim JB, Sebastiano V, Wu G, Arauzo-Bravo MJ, Sasse P (2009). Oct4-induced pluripotency in adult neural stem cells.. Cell.

[36] Maherali N, Ahfeldt T, Rigamonti A, Utikal J, Cowan C (2008). A high-efficiency system for the generation and study of human induced pluripotent stem cells.. Cell Stem Cell.

[37] Park IH, Zhao R, West JA, Yabuuchi A, Huo H (2008). Reprogramming of human somatic cells to pluripotency with defined factors.. Nature.

[38] Masaki H, Ishikawa T, Takahashi S, Okumura M, Sakai N (2007). Heterogeneity of pluripotent marker gene expression in colonies generated in human iPS cell induction culture.. Stem Cell Res.

[39] Lowry WE, Richter L, Yachechko R, Pyle AD, Tchieu J (2008). Generation of human induced pluripotent stem cells from dermal fibroblasts.. Proc Natl Acad Sci U S A.

[40] Venables WN, Ripley BD, Chambers J, Eddy W, Hardle W, Sheather S, Tierney L (2002). Modern Applied Statistics with S;.

[41] Lee JY, Ji Z, Tian B (2008). Phylogenetic analysis of mRNA polyadenylation sites reveals a role of transposable elements in evolution of the 3′-end of genes.. Nucleic Acids Res.

[42] Takagaki Y, Manley JL (1997). RNA recognition by the human polyadenylation factor CstF.. Mol Cell Biol.

[43] Perez Canadillas JM, Varani G (2003). Recognition of GU-rich polyadenylation regulatory elements by human CstF-64 protein.. EMBO J.

[44] Gangaraju VK, Lin H (2009). MicroRNAs: key regulators of stem cells.. Nat Rev Mol Cell Biol.

[45] Marson A, Levine SS, Cole MF, Frampton GM, Brambrink T (2008). Connecting microRNA genes to the core transcriptional regulatory circuitry of embryonic stem cells.. Cell.

[46] Judson RL, Babiarz JE, Venere M, Blelloch R (2009). Embryonic stem cell-specific microRNAs promote induced pluripotency.. Nat Biotechnol.

[47] Farh KK, Grimson A, Jan C, Lewis BP, Johnston WK (2005). The widespread impact of mammalian MicroRNAs on mRNA repression and evolution.. Science.

[48] Sood P, Krek A, Zavolan M, Macino G, Rajewsky N (2006). Cell-type-specific signatures of microRNAs on target mRNA expression.. Proc Natl Acad Sci U S A.

[49] Majoros WH, Ohler U (2007). Spatial preferences of microRNA targets in 3′ untranslated regions.. BMC Genomics.

[50] Chi SW, Zang JB, Mele A, Darnell RB (2009). Argonaute HITS-CLIP decodes microRNA-mRNA interaction maps.. Nature.

[51] Legendre M, Ritchie W, Lopez F, Gautheret D (2006). Differential repression of alternative transcripts: a screen for miRNA targets.. PLoS Comput Biol.

[52] Cam H, Balciunaite E, Blais A, Spektor A, Scarpulla RC (2004). A common set of gene regulatory networks links metabolism and growth inhibition.. Mol Cell.

[53] Takagaki Y, Seipelt RL, Peterson ML, Manley JL (1996). The polyadenylation factor CstF-64 regulates alternative processing of IgM heavy chain pre-mRNA during B cell differentiation.. Cell.

[54] Licatalosi DD, Mele A, Fak JJ, Ule J, Kayikci M (2008). HITS-CLIP yields genome-wide insights into brain alternative RNA processing.. Nature.

[55] Lee JY, Yeh I, Park JY, Tian B (2007). PolyA_DB 2: mRNA polyadenylation sites in vertebrate genes.. Nucleic Acids Res.

[56] Kel AE, Gossling E, Reuter I, Cheremushkin E, Kel-Margoulis OV (2003). MATCH: A tool for searching transcription factor binding sites in DNA sequences.. Nucleic Acids Res.

