# Supplementary material for: Reprogramming of 3′ Untranslated Regions of mRNAs by Alternative Polyadenylation in Generation of Pluripotent Stem Cells from Different Cell Types
Source: PLoS One. 2009 Dec 23;4(12):e8419. doi: 10.1371/journal.pone.0008419 (PMC2791866; doi:10.1371/journal.pone.0008419)
Supplement: Figure S1 — Analysis of APA using normalized Relative Usage of Distal poly(A) site score (nRUD). Method to calculate nRUD. Top, a gene with APA; Bottom, a gene without APA. CDS, coding sequence; cUTR, constitutive UTR; aUTR, alternative UTR; sUTR, single UTR; AAA, poly(A) tail. Red bars are Affymetrix (Affy) GeneChip probes. (0.02 MB PDF) [file pone.0008419.s001.pdf]

Figure S1

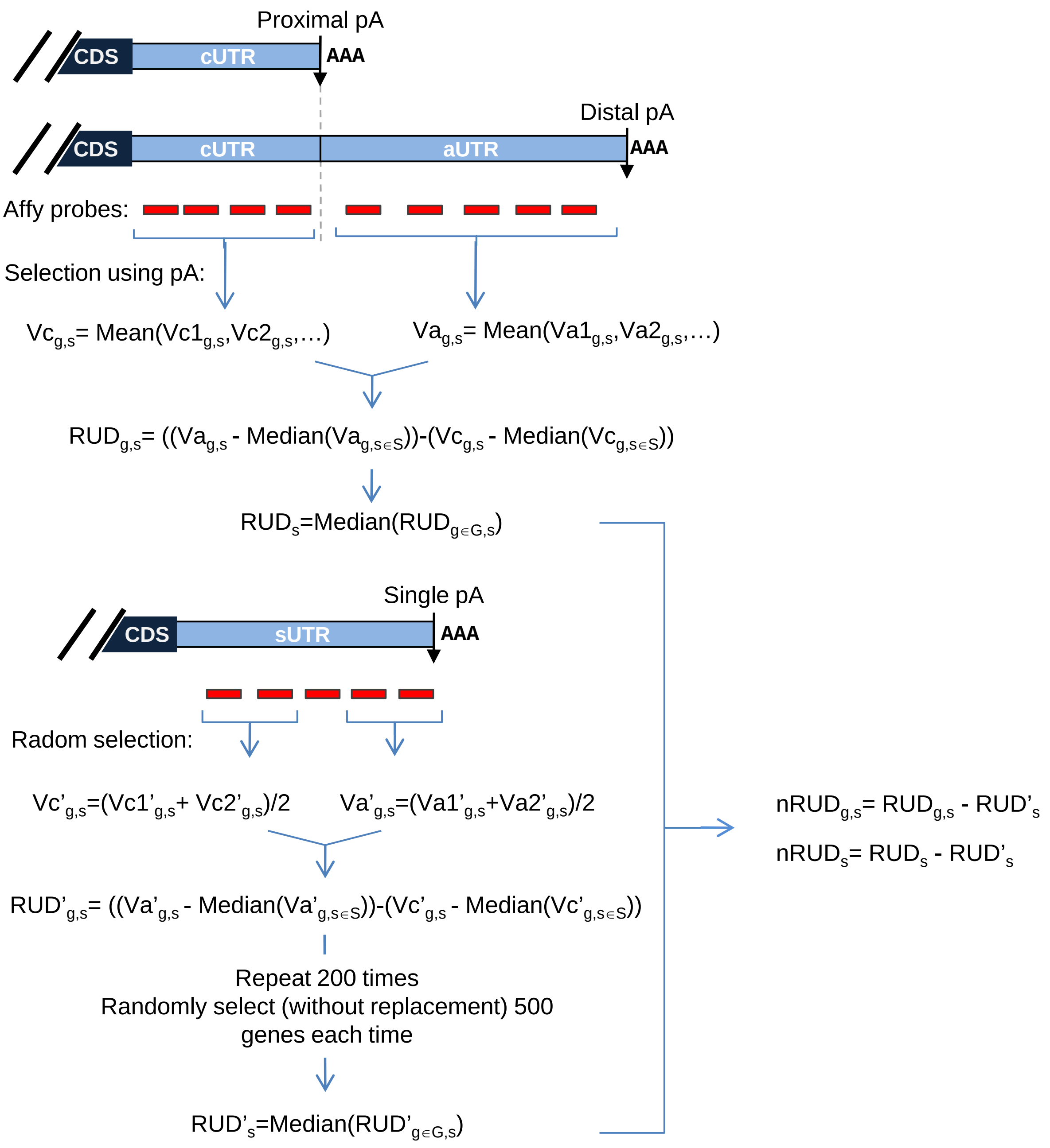

c: cUTR probe; a: aUTR probe; c': sUTR probe used as pseudo cUTR probe; a': sUTR probe used as pseudo aUTR probe ; g: gene; G: surveyed gene set; s: sample; S: sample set
Vc1, Vc2,Va1, Va2, Vc1', Vc2', Va1', and Va2': log2(probe intensity) value for a specific probe
Vc, Va, Vc' and Va': average log2(probe intensity) value for a set of probes
$RUD_{g,s}$: gene RUD, i.e. RUD value for gene g in sample s, derived from cUTR and aUTR probes
$RUD'_{g,s}$: RUD value for gene g in sample s, derived from sUTR probes
RUD_s: sample RUD, i.e. median value of $RUD_{g,s}$ for sample s
RUD'_s: expected sample RUD, i.e. median value of $RUD'_{g,s}$ for sample s
$nRUD_{g,s}$: normalized gene RUD (for gene s in sample s)
$nRUD_s$: normalized sample RUD (for sample s)
