# Supplementary figures and images for: Reprogramming of 3′ Untranslated Regions of mRNAs by Alternative Polyadenylation in Generation of Pluripotent Stem Cells from Different Cell Types
Source: PLoS One. 2009 Dec 23;4(12):e8419. doi: 10.1371/journal.pone.0008419 (PMC2791866; doi:10.1371/journal.pone.0008419)

**Figure S2**

**HU133 v2.0 (1,641 genes)**

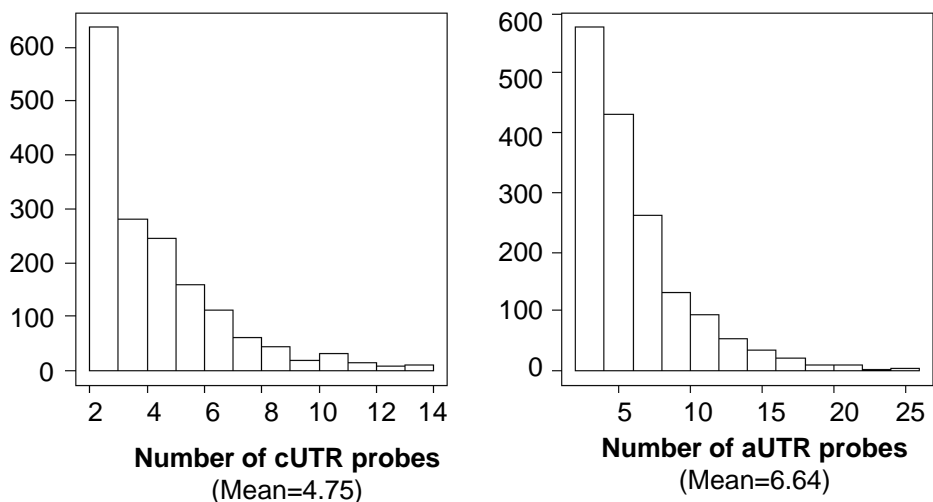

**Mouse 430 v2.0 (1,044 genes)**

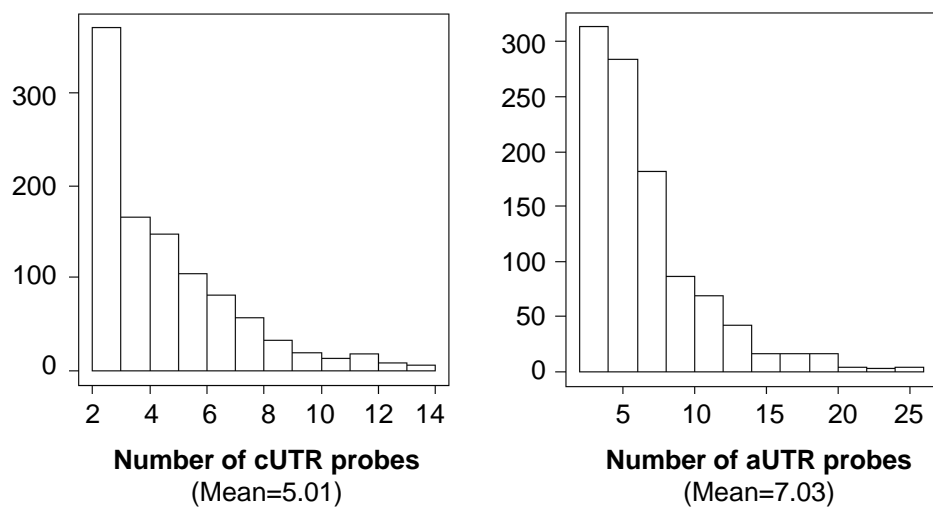

Supplement: Figure S2 — Histograms of cUTR and aUTR probe numbers for Affymetrix HU133 v2.0 GeneChip and Mouse 430 v2.0 GeneChip. All human data sets used Hu133 v2.0 and all mouse data sets used Mouse 430 v2.0. (0.01 MB PDF) [file pone.0008419.s002.pdf]

**Figure S4**

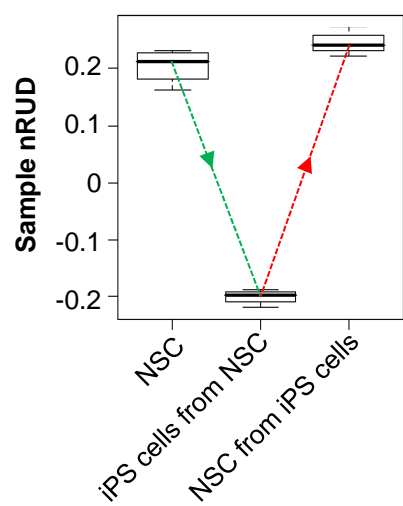

Supplement: Figure S4 — Dynamic regulation of 3′UTR by APA in generation and differentiation of iPS cells. Shortening of 3′UTRs in generation of iPS cells from adult mouse neural stem cells (NSC), and lengthening of 3′UTRs in differentiation of iPS cells to NSC. (0.02 MB PDF) [file pone.0008419.s004.pdf]

**Figure S5**

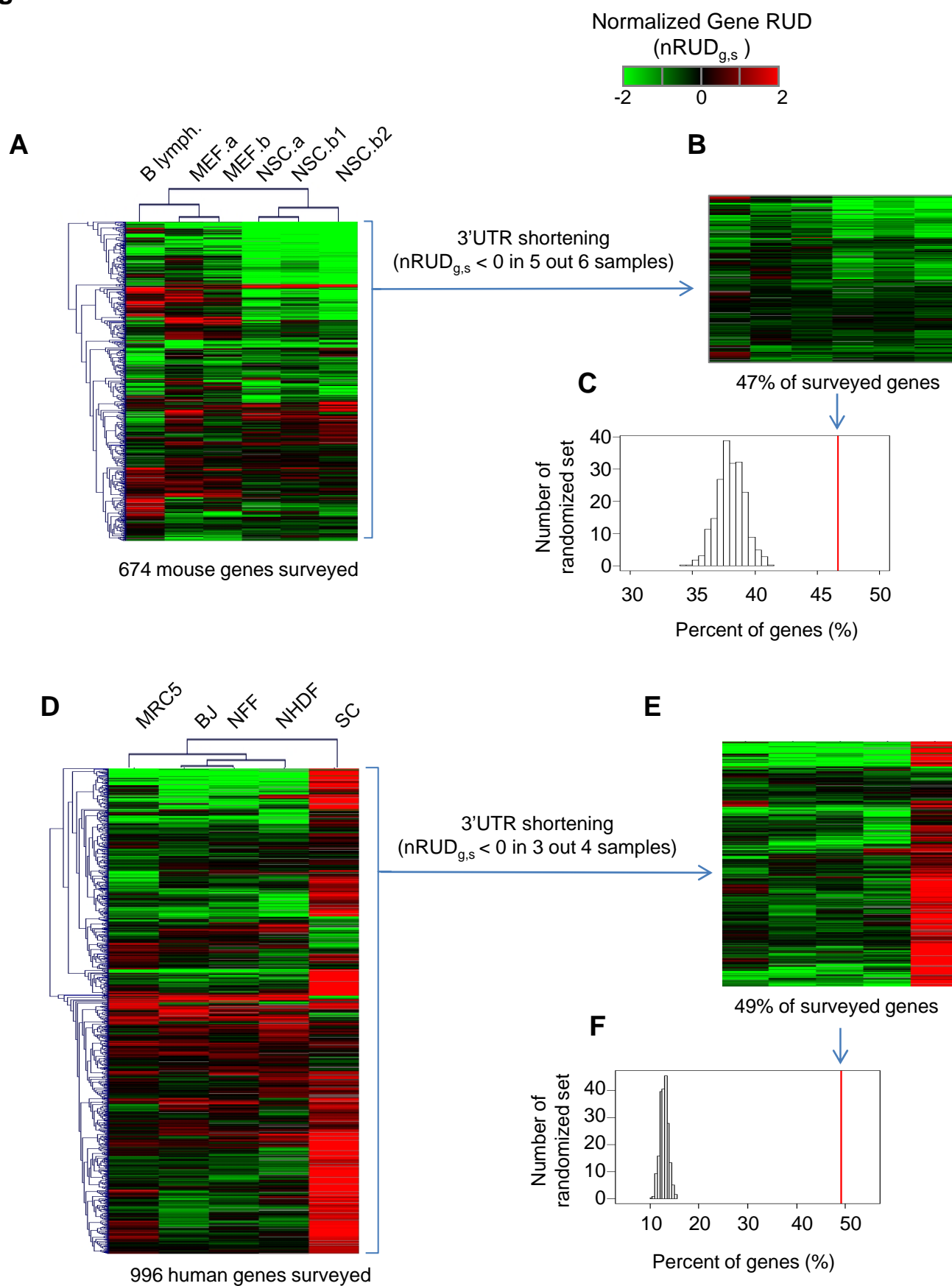

Supplement: Figure S5 — Consistent regulation of 3′UTR in generation of iPS cells across different sample sets. (A) Heatmap showing 674 mouse genes with APA surveyed in 6 sample sets. The 3′UTR regulation was measured by gene nRUD, which is represented by color according to the scale shown in the graph, with red indicating 3′UTR lengthening and green 3′UTR shortening. Samples and genes were also clustered using gene nRUD by hierarchical clustering using Pearson Correlation. (B) Genes in (A) with 3′UTR shortening in 5 out of 6 sample sets were selected (47% of total). (C) The percent of selected genes (47%) is significantly higher than expected. The histogram shows distribution of percent of genes having 3′UTR shortening in 5 out of 6 sample sets when genes are randomized in each column. The red line in the graph indicates the observed percent of genes. (D) As in (A), 996 human genes were surveyed. (E) Genes in (D) with 3′UTR shortening in 3 out 4 sample sets were selected, excluding SC. The data for SC are included for comparison. (F) As in (C), the percent of genes selected (49%) is significantly higher than that using randomized data. (0.28 MB PDF) [file pone.0008419.s005.pdf]

**Figure S7**

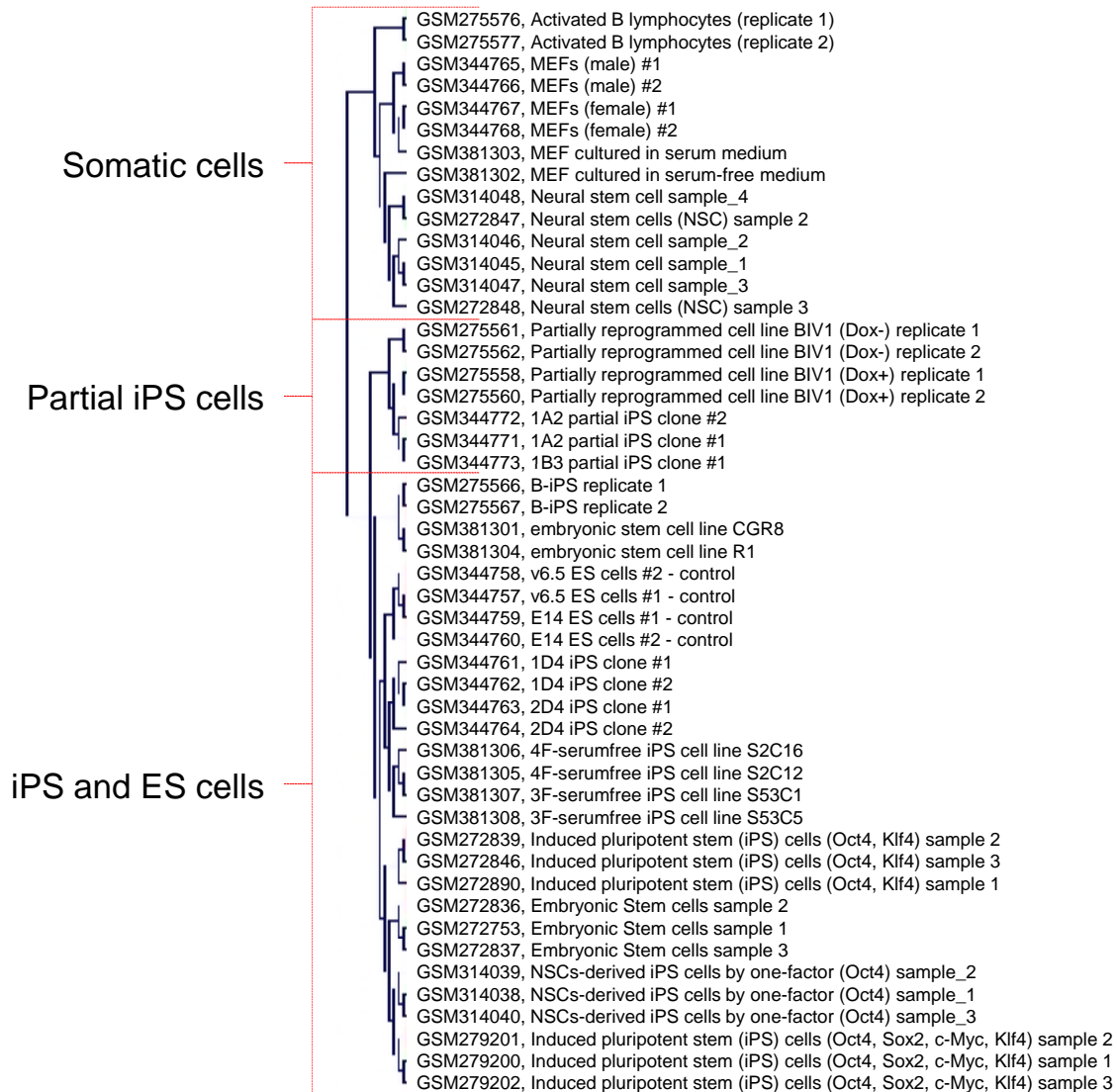

Supplement: Figure S7 — Separation of samples using gene nRUD. Sample cluster shown in Figure 2D with sample names. (0.03 MB PDF) [file pone.0008419.s007.pdf]

**Figure S8**

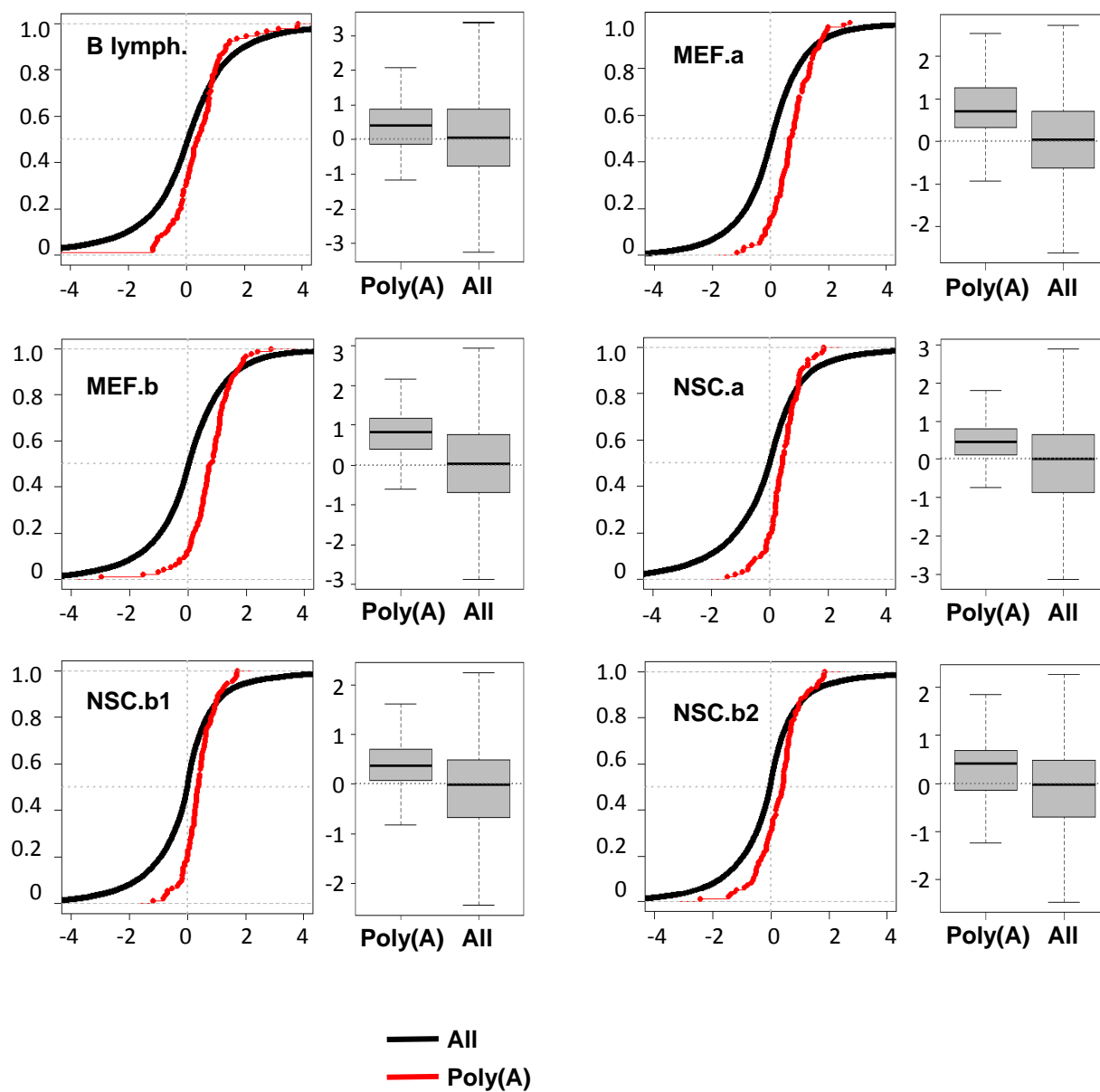

Supplement: Figure S8 — Poly(A) genes are significantly upregulated during cell reprogramming from different mouse cell types. For each data set, the ratios of gene expression after reprogramming to that before reprogramming for all genes and poly(A) genes were plotted in a cumulative distribution function (CDF) plot (left) and a boxplot (right). The difference between all genes and poly(A) genes is significant (P-value <0.005) for all data sets, based on Kolmogorov-Smironov test and Wilcoxon rank sum test. (0.05 MB PDF) [file pone.0008419.s008.pdf]

Figure S9

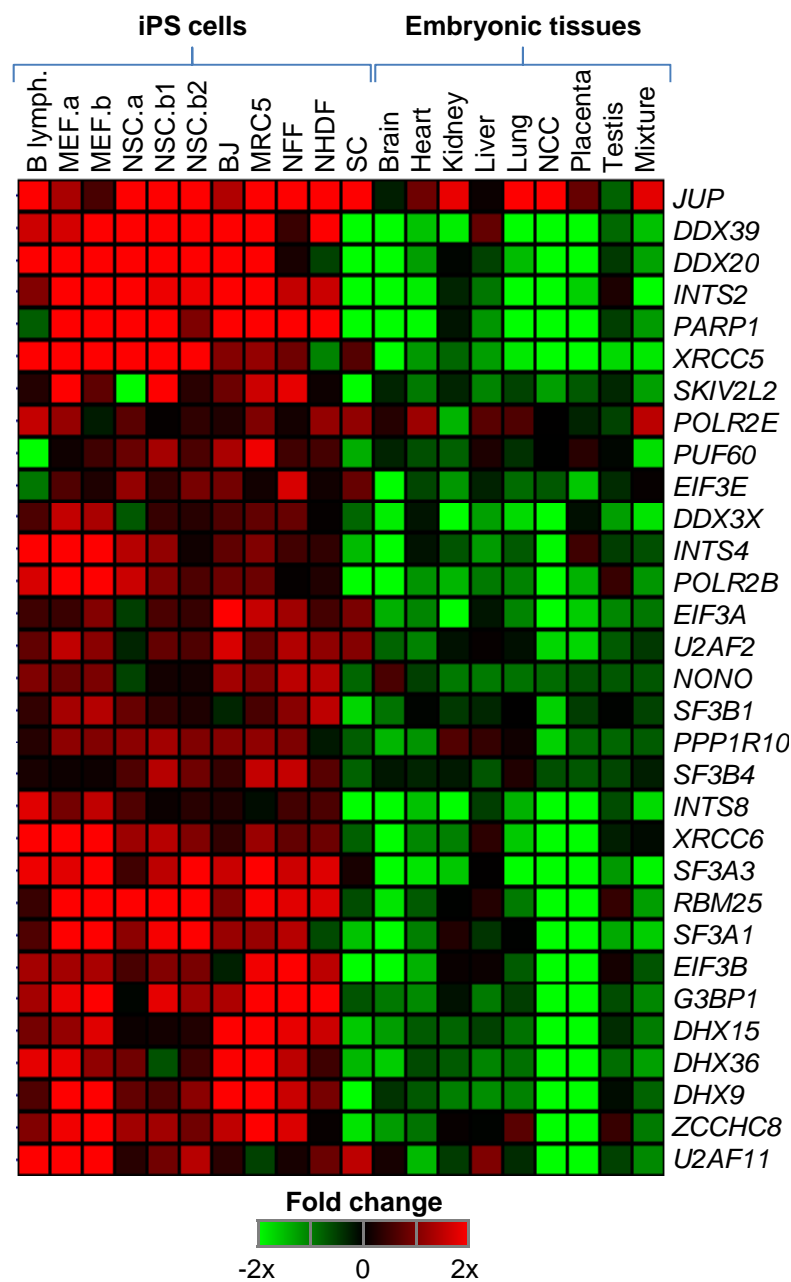

Supplement: Figure S9 — Regulation of genes encoding auxiliary polyadenylation factors in generation of iPS cells and embryonic development. For generation of iPS cells, samples before and after reprogramming were compared. Positive values indicate upregulation after reprogramming. For embryonic development, the gene expression values in the first and last days of embryonic development were compared. Samples for 8 individual tissues and mixed tissue were used, as described for Figure 5. Only the genes with consistent trend of regulation, either upregulation or downregulation in 9 out of 10 data sets for somatic cell reprogramming are shown. (0.02 MB PDF) [file pone.0008419.s009.pdf]
