# Supplementary material for: Reprogramming of 3′ Untranslated Regions of mRNAs by Alternative Polyadenylation in Generation of Pluripotent Stem Cells from Different Cell Types
Source: PLoS One. 2009 Dec 23;4(12):e8419. doi: 10.1371/journal.pone.0008419 (PMC2791866; doi:10.1371/journal.pone.0008419)

Figure S3

A

	Samples	Exp Date
1	GSM310838.CEL	3/5/2008
2	GSM310839.CEL	2/26/2008
3	GSM310844.CEL	2/26/2008
4	GSM310845.CEL	2/27/2008
5	GSM310846.CEL	2/26/2008
6	GSM310847.CEL	2/26/2008
7	GSM310848.CEL	2/26/2008
8	GSM310849.CEL	2/27/2008
9	GSM310850.CEL	2/26/2008
10	GSM310851.CEL	3/5/2008
11	GSM310852.CEL	2/26/2008
12	GSM310853.CEL	2/27/2008
13	GSM310854.CEL	5/12/2008
14	GSM310855.CEL	5/12/2008
15	GSM310856.CEL	5/13/2008
16	GSM310857.CEL	5/12/2008
17	GSM310858.CEL	5/13/2008
18	GSM310859.CEL	5/13/2008
19	GSM310860.CEL	5/13/2008
20	GSM310861.CEL	5/12/2008
21	GSM310862.CEL	5/12/2008

February/March (samples 1–12)

May (samples 13–21)

- **iPS cells**
- **Fibroblast cells**
- **ES cells**

B

C

D

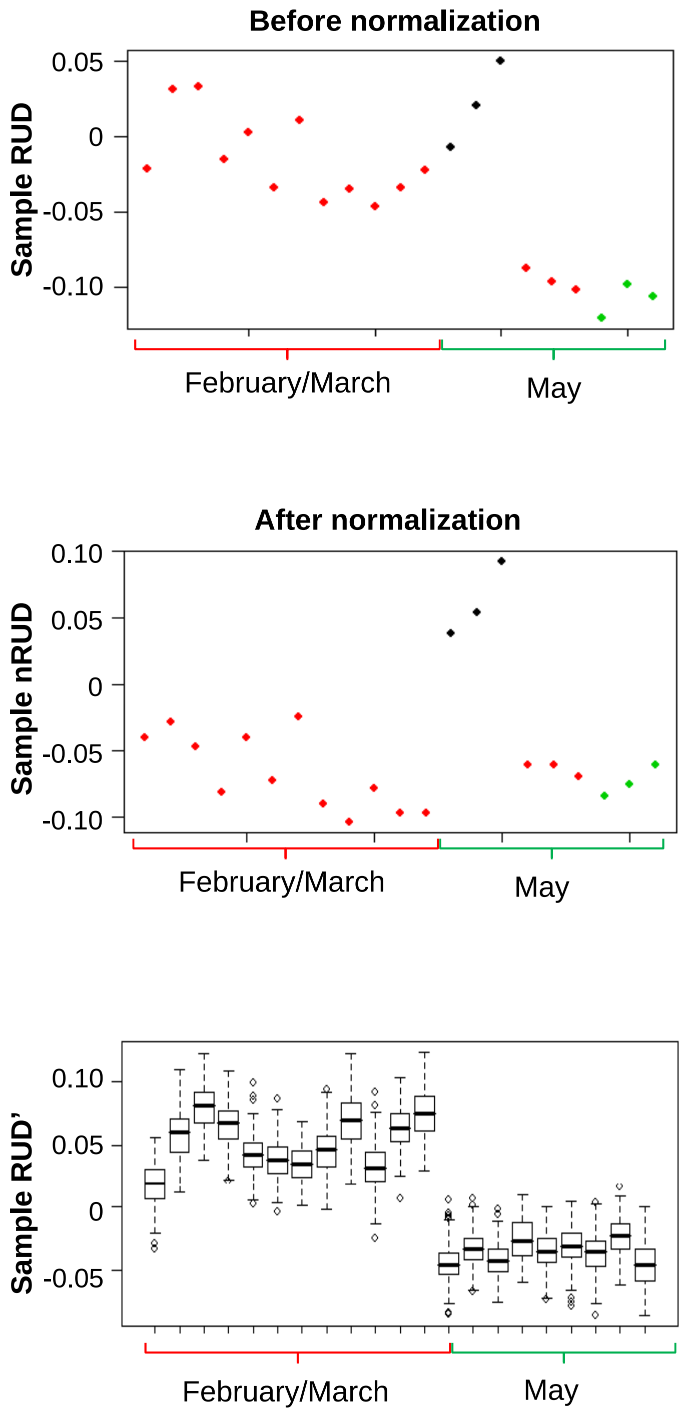

Supplement: Figure S3 — Correction of systematic differences between data sets using the nRUD method. (A) Data set for the generation of iPS cells from human BJ fibroblast (BJ in Figure 1). (B) RUD values without normalization. (C) nRUD values, i.e. RUD values with normalization. Samples in the same iPS cell group have more consistent nRUD values than original RUD values. (D) RUD' values derived from probes targeting sUTRs. The difference between February/March samples and May samples indicates systematic differences in sample processing. (0.02 MB PDF) [file pone.0008419.s003.pdf]
