# Supplementary material for: Reprogramming of 3′ Untranslated Regions of mRNAs by Alternative Polyadenylation in Generation of Pluripotent Stem Cells from Different Cell Types
Source: PLoS One. 2009 Dec 23;4(12):e8419. doi: 10.1371/journal.pone.0008419 (PMC2791866; doi:10.1371/journal.pone.0008419)

Figure S6

A

B

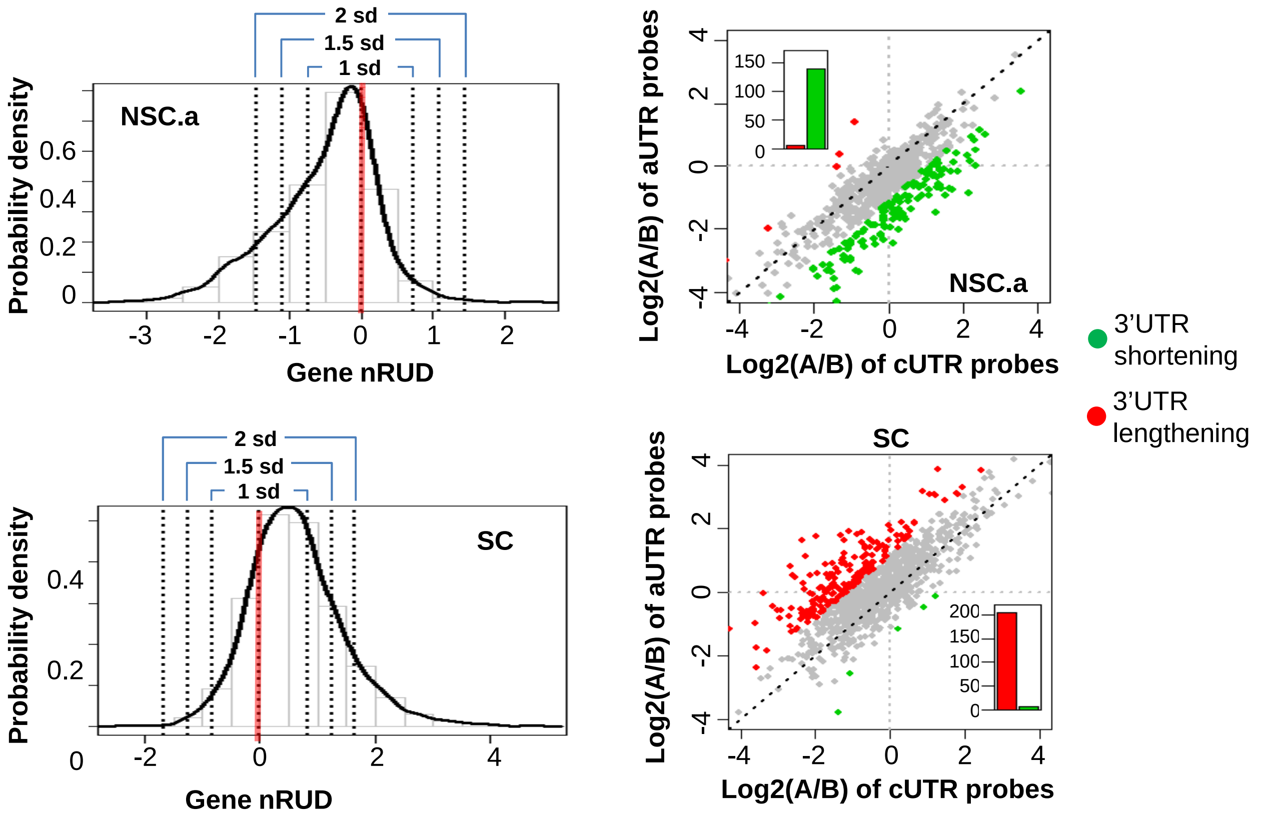

C

	1 sd			1.5 sd			2 sd		
	L	S	*P*-value	L	S	*P*-value	L	S	*P*-value
B lymph.	64	136	3.90E-04	31	67	3.55E-04	15	30	3.57E-02
MEF.a	75	111	1.01E-02	31	62	1.71E-03	11	33	1.26E-03
MEF.b	44	175	<2.2E-16	23	100	1.22E-12	12	51	7.46E-07
NSC.a	17	225	<2.2E-16	5	139	<2.2E-16	2	80	<2.2E-16
NSC.b1	16	203	<2.2E-16	9	120	<2.2E-16	3	70	<2.2E-16
NSC.b2	22	224	<2.2E-16	7	135	<2.2E-16	4	73	<2.2E-16
BJ	66	194	8.60E-16	29	103	6.47E-11	7	58	4.27E-11
MRC5	127	200	6.42E-05	59	105	4.08E-04	30	47	6.75E-02
NFF	63	167	4.79E-12	24	98	8.34E-12	9	48	1.52E-07
NHDF	58	168	1.35E-13	16	84	2.61E-12	7	51	2.40E-09
SC	359	24	<2.2E-16	204	6	<2.2E-16	108	2	<2.2E-16

L: number of genes with 3'UTR lengthening

S, number of genes with 3'UTR shortening

D

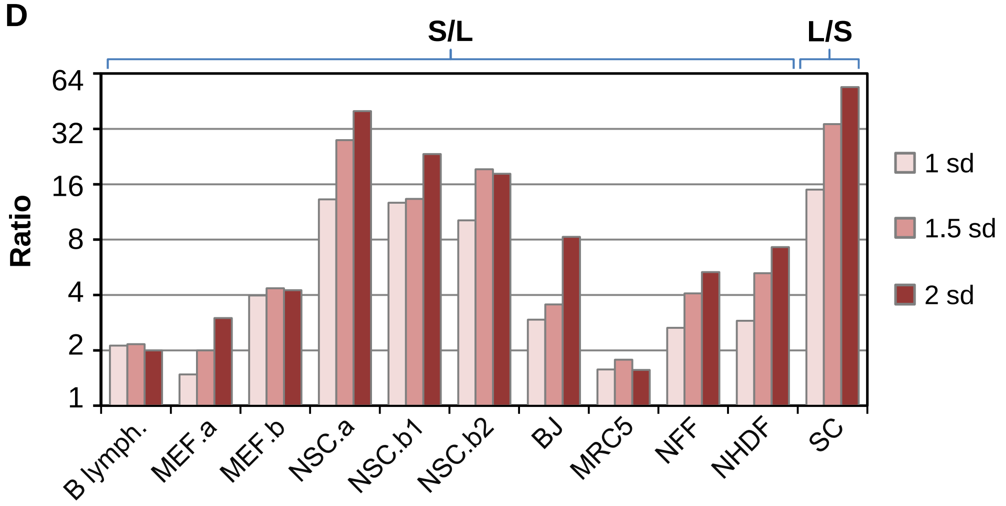

Supplement: Figure S6 — Regulation of 3′UTR in generation of iPS cells. (A) Left, distribution of nRUD for genes surveyed in NSC.a; Right, scatter plot of genes with APA. Each dot is a gene surveyed by microarray probes. X-axis and Y-axis are log2(A/B) values for probes targeting cUTRs and aUTRs, respectively, where A and B are average probe intensities for samples after and before reprogramming, respectively. Genes with nRUD greater than 1.5*(standard deviation of all genes) are shown in red, indicating significant 3′UTR lengthening, or green, indicating significant 3′UTR shortening. The numbers of genes for these 2 groups are indicated in insets. (B) Left, distribution nRUD for genes surveyed in SC; Right, scatter plot of genes with APA, as described for (A). (C) Selection of genes with 3′UTR regulation in generation of iPS cells using 3 cutoffs, i.e. 1, 1.5, and 2 standard deviation, as illustrated in (A) and (B). L and S are number of genes with 3′UTR lengthening and shortening, respectively. P-values are based on binomial tests for comparing L and S. (D) S/L and L/S are ratios. Three cutoffs were used to select genes. (0.04 MB PDF) [file pone.0008419.s006.pdf]
