# Supplementary material for: Reprogramming of 3′ Untranslated Regions of mRNAs by Alternative Polyadenylation in Generation of Pluripotent Stem Cells from Different Cell Types
Source: PLoS One. 2009 Dec 23;4(12):e8419. doi: 10.1371/journal.pone.0008419 (PMC2791866; doi:10.1371/journal.pone.0008419)

Figure S10

A. Target sites for 211 conserved miRNA families

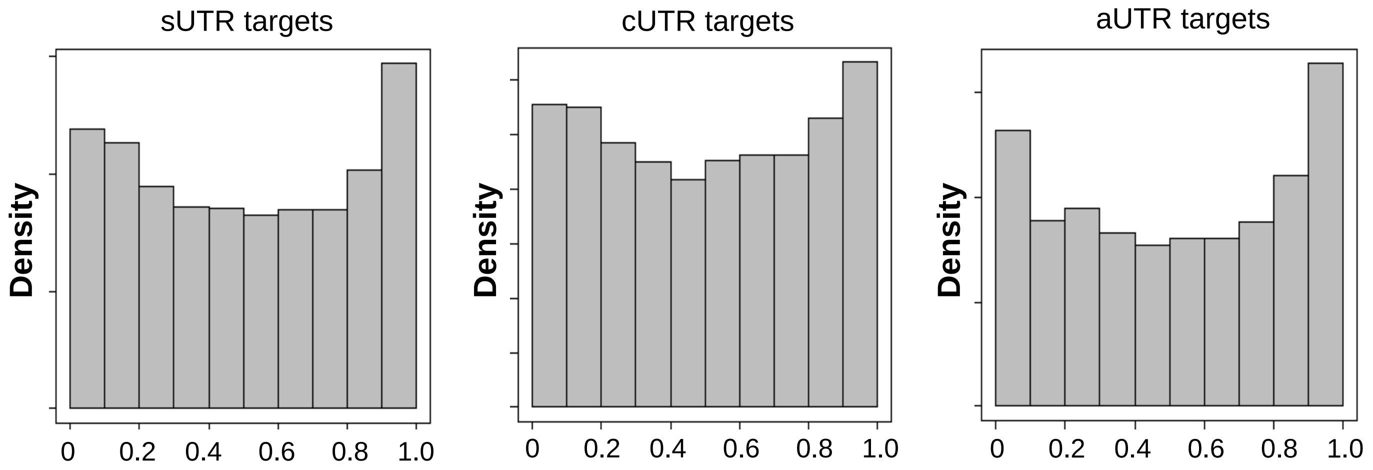

B. Target sites for 6 analyzed miRNA families

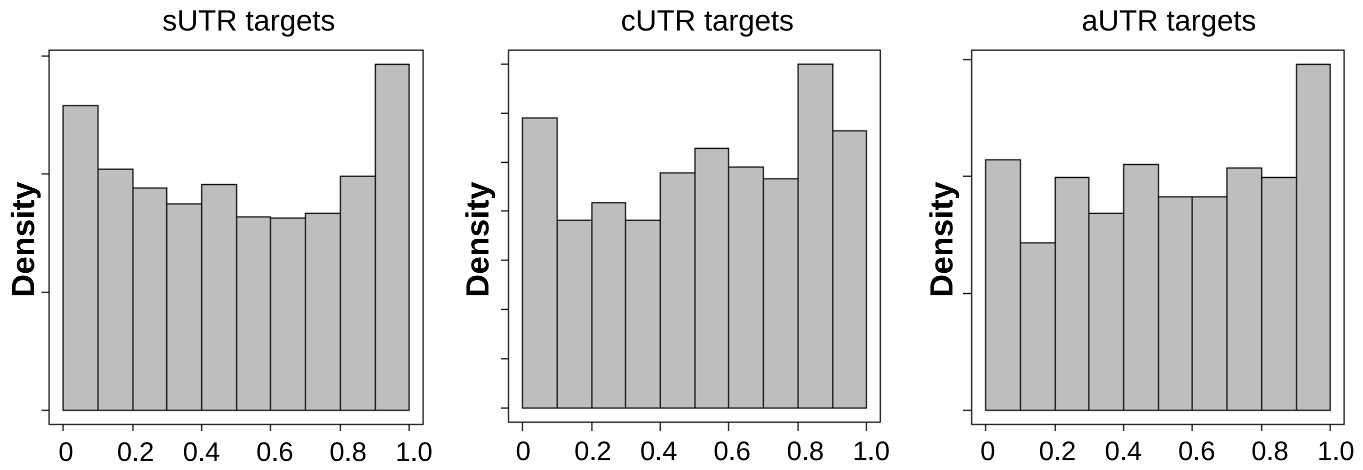

Supplement: Figure S10 — Distribution of miRNA target sites in different UTR groups. (A) Distribution of target sites for all 211 conserved miRNA families that are surveyed in this study. X-axis in each graph is relative location of target sites based on 10 evenly divided sub-regions in any given UTR. (B) Distribution of target sites for 6 miRNAs analyzed in this study. (0.01 MB PDF) [file pone.0008419.s010.pdf]
