# Supplementary material for: Reprogramming of 3′ Untranslated Regions of mRNAs by Alternative Polyadenylation in Generation of Pluripotent Stem Cells from Different Cell Types
Source: PLoS One. 2009 Dec 23;4(12):e8419. doi: 10.1371/journal.pone.0008419 (PMC2791866; doi:10.1371/journal.pone.0008419)
Supplement: Figure S11 — Transcription Factor Binding Sites (TFBS) significantly associated with RNA processing genes. P-values were based on Fisher's exact test. Sequence logos for TFBS are also shown. See Methods for detail. (0.03 MB PDF) [file pone.0008419.s011.pdf]

Figure S11

TFBS	*P*-value	Logo
E2F	$2.5x10^{-6}$	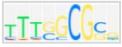
NRF-2	$1.1x10^{-4}$	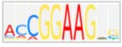
HSF	$2.0x10^{-4}$	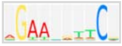
c-Myc	$1.4x10^{-3}$	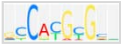
Pax-3	$2.8x10^{-3}$	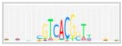
Nkx2-5	$2.9x10^{-3}$	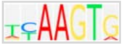
c-Rel	$5.1x10^{-3}$	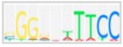
YY1	$5.0x10^{-3}$	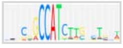
p53	$6.9x10^{-3}$	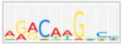
E47	$1.9x10^{-2}$	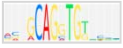
