# Supplementary material for: Reprogramming of 3′ Untranslated Regions of mRNAs by Alternative Polyadenylation in Generation of Pluripotent Stem Cells from Different Cell Types
Source: PLoS One. 2009 Dec 23;4(12):e8419. doi: 10.1371/journal.pone.0008419 (PMC2791866; doi:10.1371/journal.pone.0008419)
Supplement: Table S1 — Data sets used in this study. (0.06 MB PDF) [file pone.0008419.s012.pdf]

Table S1. Data sets used in this study.

Data Set ID	Species	Name	Cell Origin	Number of expressed genes[a]	Number of APA genes surveyed	T-test *P*-value[b]	Ref
GSE10871	Mouse	B lymph.	B lymphocyte	16,104	687	1.80E-02	[1]
GSE14012	Mouse	MEF.a	Mouse embryonic fibroblast	14,373	703	4.60E-02	[2]
GSE15267	Mouse	MEF.b	Mouse embryonic fibroblast	15,993	755	2.00E-02	-
GSE12499	Mouse	NSC.a	Adult neural stem cells	14,817	754	5.60E-06	[3]
GSE10806	Mouse	NSC.b	Adult neural stem cells	14,708	721	4.91E-04	[4]
GSE12390	Human	BJ	Neonatal foreskin fibroblast	14,755	1,078	8.56E-07	[5]
GSE9832	Human	MRC5	Fetal lung fibroblast	15,274	1,556	1.20E-02	[6]
GSE9709	Human	NFF	Neonatal foreskin fibroblast	14,903	1,036	4.10E-02	[7]
GSE9865	Human	NHDF	Neonatal foreskin fibroblast	14,280	974	2.93E-05	[8]
GSE11350	Human	SC	Spermatogonial cells from adult human testis	16,079	1,032	5.20E-06	[9]

[a]Expressed genes are those with detectable signals in at least half of samples in a set, using the MAS5 present and absent call. All mouse data sets used Affymetrix GeneChip Mouse Genome 430 v2.0, and all human data sets used Affymetrix GeneChip Human Genome U133 Plus v2.0.

[b]T-test was used to compare sample nRUD values before vs. after reprogramming, as shown in Figure 2.

1. Mikkelsen TS, Hanna J, Zhang X, Ku M, Wernig M, et al. (2008) Dissecting direct reprogramming through integrative genomic analysis. Nature 454: 49-55.
2. Sridharan R, Tchieu J, Mason MJ, Yachechko R, Kuoy E, et al. (2009) Role of the murine reprogramming factors in the induction of pluripotency. Cell 136: 364-377.
3. Kim JB, Sebastiano V, Wu G, Arauzo-Bravo MJ, Sasse P, et al. (2009) Oct4-induced pluripotency in adult neural stem cells. Cell 136: 411-419.
4. Kim JB, Zaehres H, Wu G, Gentile L, Ko K, et al. (2008) Pluripotent stem cells induced from adult neural stem cells by reprogramming with two factors. Nature 454: 646-650.
5. Maherali N, Ahfeldt T, Rigamonti A, Utikal J, Cowan C, et al. (2008) A high-efficiency system for the generation and study of human induced pluripotent stem cells. Cell Stem Cell 3: 340-345.
6. Park IH, Zhao R, West JA, Yabuuchi A, Huo H, et al. (2008) Reprogramming of human somatic cells to pluripotency with defined factors. Nature 451: 141-146.
7. Masaki H, Ishikawa T, Takahashi S, Okumura M, Sakai N, et al. (2007) Heterogeneity of pluripotent marker gene expression in colonies generated in human iPS cell induction culture. Stem Cell Res 1: 105-115.
8. Lowry WE, Richter L, Yachechko R, Pyle AD, Tchieu J, et al. (2008) Generation of human induced pluripotent stem cells from dermal fibroblasts. Proc Natl Acad Sci U S A 105: 2883-2888.
9. Conrad S, Renninger M, Hennenlotter J, Wiesner T, Just L, et al. (2008) Generation of pluripotent stem cells from adult human testis. Nature 456: 344-349.
