# Supplementary material for: Reprogramming of 3′ Untranslated Regions of mRNAs by Alternative Polyadenylation in Generation of Pluripotent Stem Cells from Different Cell Types
Source: PLoS One. 2009 Dec 23;4(12):e8419. doi: 10.1371/journal.pone.0008419 (PMC2791866; doi:10.1371/journal.pone.0008419)
Supplement: Table S2 — Microarray samples used in this study. (0.02 MB PDF) [file pone.0008419.s013.pdf]

Table S2. Microarray samples used in this study.

Sample ID	Sample name	Group
B lymph., GSE10871		
GSM275576	Activated B lymphocytes (replicate 1)	B
GSM275577	Activated B lymphocytes (replicate 2)	B
GSM275558	Partially reprogrammed cell line BIV1 (Dox+) replicate 1	P1
GSM275560	Partially reprogrammed cell line BIV1 (Dox+) replicate 2	P1
GSM275561	Partially reprogrammed cell line BIV1 (Dox-) replicate 1	P2
GSM275562	Partially reprogrammed cell line BIV1 (Dox-) replicate 2	P2
GSM275566	B-iPS replicate 1	A
GSM275567	B-iPS replicate 2	A
MEF.a, GSE14012		
GSM344765	MEFs (male) #1	B
GSM344766	MEFs (male) #2	B
GSM344767	MEFs (female) #1	B
GSM344768	MEFs (female) #2	B
GSM344771	1A2 partial iPS clone #1	P
GSM344772	1A2 partial iPS clone #2	P
GSM344773	1B3 partial iPS clone #1	P
GSM344761	1D4 iPS clone #1	A
GSM344762	1D4 iPS clone #2	A
GSM344763	2D4 iPS clone #1	A
GSM344764	2D4 iPS clone #2	A
GSM344757	v6.5 ES cells #1 – control	E
GSM344758	v6.5 ES cells #2 – control	E
GSM344759	E14 ES cells #1 – control	E
GSM344760	E14 ES cells #2 – control	E
MEF.b, GSE15267		
GSM381302	MEF cultured in serum-free medium	B
GSM381303	MEF cultured in serum medium	B
GSM381305	4F-serumfree iPS cell line S2C12	A
GSM381306	4F-serumfree iPS cell line S2C16	A
GSM381307	3F-serumfree iPS cell line S53C1	A
GSM381308	3F-serumfree iPS cell line S53C5	A
GSM381304	embryonic stem cell line R1	E
GSM381301	embryonic stem cell line CGR8	E
NSC.a, GSE12499		
GSM314038	NSCs-derived iPS cells by one-factor (Oct4) sample_1	A
GSM314039	NSCs-derived iPS cells by one-factor (Oct4) sample_2	A
GSM314040	NSCs-derived iPS cells by one-factor (Oct4) sample_3	A
GSM314045	Neural stem cell sample_1	B
GSM314046	Neural stem cell sample_2	B
GSM314047	Neural stem cell sample_3	B
GSM314048	Neural stem cell sample_4	B
NSC.b, GSE10806		
GSM272847	Neural stem cells (NSC) sample 2	B
GSM272848	Neural stem cells (NSC) sample 3	B
GSM272839	Induced pluripotent stem (iPS) cells (Oct4, Klf4) sample 2	A1
GSM272846	Induced pluripotent stem (iPS) cells (Oct4, Klf4) sample 3	A1
GSM272890	Induced pluripotent stem (iPS) cells (Oct4, Klf4) sample 1	A1
GSM279200	Induced pluripotent stem (iPS) cells (Oct4, Sox2, c-Myc, Klf4) sample 1	A2
GSM279201	Induced pluripotent stem (iPS) cells (Oct4, Sox2, c-Myc, Klf4) sample 2	A2
GSM279202	Induced pluripotent stem (iPS) cells (Oct4, Sox2, c-Myc, Klf4) sample 3	A2
GSM272753	Embryonic Stem cells sample 1	E
GSM272836	Embryonic Stem cells sample 2	E
GSM272837	Embryonic Stem cells sample 3	E
BJ, GSE12390		
GSM310854	BJ sample 1	B
GSM310855	BJ sample 2	B
GSM310856	BJ sample 3	B
GSM310838	BJ hIPS #5p9 sample 1	A
GSM310839	BJ hIPS #5p9 sample 2	A
GSM310844	BJ hIPS #5p9 sample 3	A
GSM310845	BJ hIPS #6p9 sample 1	A
GSM310846	BJ hIPS #6p9 sample 2	A

GSM310847	BJ hIPS #6p9 sample 3	A
GSM310848	BJ hIPS #8p10 sample 1	A
GSM310849	BJ hIPS #8p10 sample 2	A
GSM310850	BJ hIPS #8p10 sample 3	A
GSM310851	BJ hIPS #12p5 sample 1	A
GSM310852	BJ hIPS #12p5 sample 2	A
GSM310853	BJ hIPS #12p5 sample 3	A
GSM310857	BJ hIPS #12p6 afp 4 #12 p 7 sample 1	A
GSM310858	BJ hIPS #12p6 afp 4 #12 p 7 sample 2	A
GSM310859	BJ hIPS #12p6 afp 4 #12 p 7 sample 3	A
GSM310860	HUES 8 p 30 sample 1	E
GSM310861	HUES 8 p 30 sample 2	E
GSM310862	HUES 8 p 30 sample 3	E
NHDF, GSE9865		
GSM249026	Fibroblasts with GFP virus NHDF1 + GFP	B
GSM249027	Fibroblasts NHDF1	B
GSM249029	Fibroblasts and 5 factors after 18 days NHDF1 +5V	B
GSM249150	Partially reprgrammed clone Hips24	P
GSM249151	Partially reprogrammed clone Clone100	P
GSM249152	Partially reprogrammed clone Hips29	P
GSM249028	Reprogrammed clone Hips1	A
GSM249095	Reprogrammed clone 5 hips5	A
GSM249096	Reprogrammed clone 2 Hips2	A
GSM249137	Reprogrammed clone 7 Hips7	A
GSM249282	Human embryonic stem cell line H9	E
GSM249025	Human Embryonic Stem Cells passage 49 HSF1	E
MRC5, GSE9832		
GSM248209	MRC5 fibroblast_40	B
GSM248210	MRC5 fibroblast_59	B
GSM248211	MRC5-iPS2 iPS cells_2	A
GSM248212	MRC5-iPS2 iPS cells_22	A
NFF, GSE9709		
GSM257524	Human neonatal dermal fibroblast (5F0416)	B
GSM245341	Human neonatal dermal fibroblast (5F0438)	B
GSM245339	Human induced pluripotent stem cell clone 1-8 cultured in mTeSR1 on Matrigel (2)	A
GSM245342	Human induced pluripotent stem cell clone 1-8 cultured in MEF-conditioned medium on Matrigel	A
GSM248216	Human induced pluripotent stem cell clone 1-8 cultured in mTeSR1 on Matrigel (1)	A
GSM248217	Human induced pluripotent stem cell clone 1-8 cultured in ESM on MEF	A
GSM257520	Human induced pluripotent stem cell clone 1-8 cultured in mTeSR1 on Matrigel after freeze-thaw treatment	A
GSM257521	Human induced pluripotent stem cell clone 2-4 cultured in mTeSR1 on Matrigel	A
GSM257522	Human induced pluripotent stem cell clone 2-4 cultured in ESM on MEF	A
GSM257523	Human induced pluripotent stem cell clone 3-2 cultured in mTeSR1 on Matrigel	A
SC, GSE11350		
GSM282008	Generation of pluripotent stem cells from adult human testis_hGS1	B
GSM282012	Generation of pluripotent stem cells from adult human testis_hGS2	B
GSM282013	Generation of pluripotent stem cells from adult human testis_hGS3	B
GSM282014	Generation of pluripotent stem cells from adult human testis_haGSC4	A
GSM282015	Generation of pluripotent stem cells from adult human testis_haGSC5	A
GSM282016	Generation of pluripotent stem cells from adult human testis_haGSC6	A
GSM282017	Generation of pluripotent stem cells from adult human testis_haGSC1	A
GSM282018	Generation of pluripotent stem cells from adult human testis_haGSC2	A
GSM282019	Generation of pluripotent stem cells from adult human testis_haGSC3	A
GSM282009	Generation of pluripotent stem cells from adult human testis_ES1	E
GSM282010	Generation of pluripotent stem cells from adult human testis_ES2	E
GSM282011	Generation of pluripotent stem cells from adult human testis_ES3	E

Sample ID, NCBI GEO sample ID; Sample name, NCBI GEO sample name; Group, sample group used in Figure 1. Data set name used in this study (Table S1) and NCBI GEO data set ID are shown for each data set.
