# Supplementary material for: Reprogramming of 3′ Untranslated Regions of mRNAs by Alternative Polyadenylation in Generation of Pluripotent Stem Cells from Different Cell Types
Source: PLoS One. 2009 Dec 23;4(12):e8419. doi: 10.1371/journal.pone.0008419 (PMC2791866; doi:10.1371/journal.pone.0008419)
Supplement: Table S3 — Poly(A) genes analyzed in this study. (0.11 MB PDF) [file pone.0008419.s014.pdf]

Table S3. Poly(A) genes analyzed in this study.

Hs.Sym	Mm.Sym	Human133v2	Mouse430v2	Type	Gene Name
PPP1CB	*Ppp1cb*	201409_s_at	1431328_at	c	protein phosphatase 1, catalytic subunit, beta isoform
CSTF2	*Cstf2*	204459_at	1419644_at	c	Cleavage stimulation factor, 3' pre-RNA, subunit 2, 64kDa
CSTF3	*Cstf3*	203947_at	1424723_s_at	c	cleavage stimulation factor, 3' pre-RNA, subunit 3, 77kDa
CPSF1	*Cpsf1*	33132_at	1417665_a_at	c	cleavage and polyadenylation specific factor 1, 160kDa
WDR33	*Wdr33*	223147_s_at	1453554_a_at	c	WD repeat domain 33
CPSF6	*Cpsf6*	202469_s_at	1428232_at	c	cleavage and polyadenylation specific factor 6, 68kDa
FIP1L1	*Fip1l1*	1554424_at	1452720_a_at	c	FIP1 like 1 (S. cerevisiae)
NUDT21	*Nudt21*	202697_at	1417681_at	c	nudix (nucleoside diphosphate linked moiety X)-type motif 21
PABPC4	*Pabpc4*	201064_s_at	1421046_a_at	c	poly(A) binding protein, cytoplasmic 4 (inducible form)
FLJ12529	*5730453I16Rik*	217866_at	1453356_at	c	pre-mRNA cleavage factor I, 59 kDa subunit
PCF11	*Pcf11*	203378_at	1427159_at	c	PCF11, cleavage and polyadenylation factor subunit, homolog (S. cerevisiae)
CSTF1	*Cstf1*	202190_at	1448597_at	c	cleavage stimulation factor, 3' pre-RNA, subunit 1, 50kDa
LOC341315	*Pabpc1*	215823_x_at	1418883_a_at	c	poly(A) binding protein, cytoplasmic 3
CPSF4	*Cpsf4*	206688_s_at	1424469_a_at	c	cleavage and polyadenylation specific factor 4, 30kDa
CSTF2T	*Cstf2t*	212901_s_at	1415920_at	c	cleavage stimulation factor, 3' pre-RNA, subunit 2, 64kDa, tau variant
CPSF3	*Cpsf3*	225082_at	1437852_x_at	c	cleavage and polyadenylation specific factor 3, 73kDa
CPSF2	*Cpsf2*	225994_at	1420937_at	c	cleavage and polyadenylation specific factor 2, 100kDa
PAPOLG	*Papolg*	224427_s_at	1427241_at	c	poly(A) polymerase gamma
CLP1	*Clp1*	229496_at	1423612_at	c	CLP1, cleavage and polyadenylation factor I subunit, homolog (S. cerevisiae)
PPP1CA	*Ppp1ca*	200846_s_at	1460165_at	c	protein phosphatase 1, catalytic subunit, alpha isoform
PABPN1	*Pabpn1*	201544_x_at	1422849_a_at	c	poly(A) binding protein, nuclear 1
SYMPK	*Sympk*	32402_s_at	1428673_at	c	symplekin
RBBP6	*Rbbp6*	212783_at	1426487_a_at	c	retinoblastoma binding protein 6
INTS8	*Ints8*	218905_at	1431096_at	a	integrator complex subunit 8
SFPQ	*Sfpq*	201585_s_at	1438459_x_at	a	Splicing factor proline/glutamine-rich (polypyrimidine tract binding protein associated)
DHX36	*Dhx36*	223140_s_at	1424397_at	a	DEAH (Asp-Glu-Ala-His) box polypeptide 36
POLR2B	*Polr2b*	201803_at	1433552_a_at	a	polymerase (RNA) II (DNA directed) polypeptide B, 140kDa
PRPF19	*Prpf19*	203103_s_at	1456531_x_at	a	PRP19/PSO4 pre-mRNA processing factor 19 homolog (S. cerevisiae)
IFIT1	*2010002M12Rik*	203153_at	1457976_at	a	interferon-induced protein with tetratricopeptide repeats 1
RBM7	*Rbm7*	235045_at	1451237_s_at	a	RNA binding motif protein 7
TAF15	*Taf15*	202840_at	1453504_at	a	TAF15 RNA polymerase II, TATA box binding protein (TBP)-associated factor, 68kDa
SKIV2L2	*Skiv2l2*	227447_at	1426718_at	a	superkiller viralicidic activity 2-like 2 (S. cerevisiae)
SRRM2	*Srrm2*	208610_s_at	1437638_at	a	Serine/arginine repetitive matrix 2
EIF3H	*Eif3h*	201592_at	1434324_x_at	a	eukaryotic translation initiation factor 3, subunit H
EIF3I	*Eif3i*	208756_at	1416233_at	a	eukaryotic translation initiation factor 3, subunit I

SNRP70	*Snrp70*	213121_at	1451104_a_at	a	small nuclear ribonucleoprotein 70kDa polypeptide (RNP antigen)
RBM9	*Rbm9*	216215_s_at	1434938_at	a	RNA binding motif protein 9
DDX20	*Ddx20*	223331_s_at	1416751_a_at	a	DEAD (Asp-Glu-Ala-Asp) box polypeptide 20
INTS7	*Ints7*	222250_s_at	1452822_at	a	integrator complex subunit 7
PPP1R10	*LOC677319*	201702_s_at	1426726_at	a	protein phosphatase 1, regulatory (inhibitor) subunit 10
EIF3F	*Eif3f*	200023_s_at	1452285_a_at	a	eukaryotic translation initiation factor 3, subunit F
G3BP1	*G3bp1*	201514_s_at	1422801_at	a	GTPase activating protein (SH3 domain) binding protein 1
BAT1	*Bat1a*	200041_s_at	1437984_x_at	a	HLA-B associated transcript 1
INTS9	*Ints9*	237286_at	1436896_at	a	Integrator complex subunit 9
INTS6	*Ints6*	218819_at	1423274_at	a	integrator complex subunit 6
DHX15	*Dhx15*	201385_at	1416145_at	a	DEAH (Asp-Glu-Ala-His) box polypeptide 15
INTS10	*Ints10*	229633_at	1452810_at	a	integrator complex subunit 10
DDX6	*Ddx6*	204909_at	1434697_at	a	DEAD (Asp-Glu-Ala-Asp) box polypeptide 6
XRCC5	*Xrcc5*	208642_s_at	1451968_at	a	X-ray repair complementing defective repair in Chinese hamster cells 5 (double-strand-break rejoining; Ku autoantigen, 80kDa)
SF3B2	*Sf3b2*	200619_at	1453123_at	a	splicing factor 3b, subunit 2, 145kDa
RBM39	*Rbm39*	238357_at	1442744_at	a	RNA binding motif protein 39
JUP	*Jup*	201015_s_at	1426873_s_at	a	junction plakoglobin
U2AF1	*U2af1*	202858_at	1422509_at	a	U2 small nuclear RNA auxiliary factor 1
XRCC6	*Xrcc6*	200792_at	1417437_at	a	X-ray repair complementing defective repair in Chinese hamster cells 6 (Ku autoantigen, 70kDa)
CDC73	*Cdc73*	218578_at	1427971_at	a	cell division cycle 73, Paf1/RNA polymerase II complex component, homolog (S. cerevisiae)
DDX5	*Ddx5*	200033_at	1433810_x_at	a	DEAD (Asp-Glu-Ala-Asp) box polypeptide 5
SF3B14	*0610009D07Rik*	1561923_a_at	1436681_x_at	a	splicing factor 3B, 14 kDa subunit
INTS4	*Ints4*	225169_at	1423806_at	a	integrator complex subunit 4
SF3A1	*Sf3a1*	216457_s_at	1449333_at	a	splicing factor 3a, subunit 1, 120kDa
EIF3A	*Eif3s10*	200596_s_at	1448425_at	a	eukaryotic translation initiation factor 3, subunit A
RBM25	*Rbm25*	212031_at	1428908_at	a	RNA binding motif protein 25
SF3A3	*Sf3a3*	203818_s_at	1423811_at	a	splicing factor 3a, subunit 3, 60kDa
U2AF2	*U2af2*	218382_s_at	1417260_at	a	U2 small nuclear RNA auxiliary factor 2
EEF1G	*Eef1g*	200689_x_at	1458955_at	a	eukaryotic translation elongation factor 1 gamma
POLR2E	*Polr2e*	217854_s_at	1417138_s_at	a	polymerase (RNA) II (DNA directed) polypeptide E, 25kDa
EEF1A1	*Eef1a1*	227708_at	1424635_at	a	eukaryotic translation elongation factor 1 alpha 1
CD68	*Eif4a1*	203507_at	1427058_at	a	Eukaryotic translation initiation factor 4A, isoform 1
DDX39	*Ddx39*	201584_s_at	1423643_at	a	DEAD (Asp-Glu-Ala-Asp) box polypeptide 39
EIF4G1	*Eif4g1*	208624_s_at	1438686_at	a	eukaryotic translation initiation factor 4 gamma, 1
SND1	*Snd1*	201622_at	1416038_at	a	staphylococcal nuclease and tudor domain containing 1
GNB2L1	*Gnb2l1*	222034_at	1455168_a_at	a	Guanine nucleotide binding protein (G protein), beta polypeptide 2-like 1
SF3B1	*Sf3b1*	201070_x_at	1449138_at	a	splicing factor 3b, subunit 1, 155kDa
INTS3	*Ints3*	211132_at	1423922_s_at	a	integrator complex subunit 3
PRPF38B	*Prpf38b*	230270_at	1455016_at	a	PRP38 pre-mRNA processing factor 38 (yeast) domain containing B

GTF2I	*Gtf2i*	210892_s_at	1425628_a_at	a	general transcription factor II, i
SF3B4	*Sf3b4*	209044_x_at	1424619_at	a	splicing factor 3b, subunit 4, 49kDa
SF3A2	*Sf3a2*	37462_i_at	1455546_s_at	a	splicing factor 3a, subunit 2, 66kDa
SF1	*Sf1*	210172_at	1423750_a_at	a	splicing factor 1
EIF2A	*Eif2a*	223015_at	1460575_at	a	eukaryotic translation initiation factor 2A, 65kDa
SUPT16H	*Supt16h*	217815_at	1449578_at	a	suppressor of Ty 16 homolog (S. cerevisiae)
INTS2	*Ints2*	224308_s_at	1436247_at	a	integrator complex subunit 2
EIF3B	*Eif3b*	211501_s_at	1426674_at	a	eukaryotic translation initiation factor 3, subunit B
POLR2A	*Polr2a*	217420_s_at	1426242_at	a	polymerase (RNA) II (DNA directed) polypeptide A, 220kDa
IFIT3	*Ifit3*	229450_at	1449025_at	a	interferon-induced protein with tetratricopeptide repeats 3
PRKDC	*Prkdc*	208694_at	1451576_at	a	Protein kinase, DNA-activated, catalytic polypeptide
ZCCHC8	*Zcchc8*	218478_s_at	1423755_at	a	zinc finger, CCHC domain containing 8
PARP1	*Parp1*	208644_at	1422503_s_at	a	poly (ADP-ribose) polymerase family, member 1
DDX23	*Ddx23*	201440_at	1430050_at	a	DEAD (Asp-Glu-Ala-Asp) box polypeptide 23
DHX9	*Dhx9*	202420_s_at	1425617_at	a	DEAH (Asp-Glu-Ala-His) box polypeptide 9
EIF3E	*Eif3e*	208697_s_at	1434523_x_at	a	eukaryotic translation initiation factor 3, subunit E
NONO	*Nono*	210470_x_at	1447160_at	a	non-POU domain containing, octamer-binding
DDX3X	*Ddx3x*	212514_x_at	1416467_at	a	DEAD (Asp-Glu-Ala-Asp) box polypeptide 3, X-linked
PUF60	*Puf60*	209899_s_at	1430705_at	a	poly-U binding splicing factor 60KDa
DDX17	*Ddx17*	213998_s_at	1439037_at	a	DEAD (Asp-Glu-Ala-Asp) box polypeptide 17

The list is largely based on a recent proteomics study of the polyadenylation machinery [1]. Only genes having both human and mouse orthologs in the HomoloGene database were analyzed in this study. Hs.Sym, human gene symbol in NCBI Gene database; Mm.Sym, mouse gene symbol in NCBI Gene database; Human133v2, human Genome U133 Plus v2.0 GeneChip probesets used for analysis; Mouse 430v2, mouse Genome 430 v2.0 GeneChip probesets used for analysis; Type, core (c) or associated/auxiliary (a) polyadenylation factors; Gene Name, gene name obtained from NCBI Gene database. When a gene has multiple probe sets, we select the one that is not affected by APA and/or most consistent across data sets with respect to direction of gene regulation.

1. Shi Y, Di Giammartino DC, Taylor D, Sarkeshik A, Rice WJ, et al. (2009) Molecular architecture of the human pre-mRNA 3' processing complex. Mol Cell 33: 365-376.
