# Supplementary material for: Reprogramming of 3′ Untranslated Regions of mRNAs by Alternative Polyadenylation in Generation of Pluripotent Stem Cells from Different Cell Types
Source: PLoS One. 2009 Dec 23;4(12):e8419. doi: 10.1371/journal.pone.0008419 (PMC2791866; doi:10.1371/journal.pone.0008419)
Supplement: Table S4 — Prediction of function in generation of iPS cells for 211 miRNA families. (0.05 MB PDF) [file pone.0008419.s015.pdf]

Table S4. Prediction of function in generation of iPS cells for 211 miRNA families.

miRNA family	Seed	B lymph.	MEF.a	MEF.b	NSC.a	NSC.b1	NSC.b2	BJ	MRC5	NFF	NHDF	SC	Mean
miR-124/506	**AAGGCAC**	-13.5	-22.2	-14.2	-26.5	-27.8	-28.2	-5.2	-8.5	-9.0	-9.3	6.3	**-14.4**
miR-291b-3p/519a/519b-3p/519c-3p	**AAGUGCA**	-6.2	-11.6	-10.7	-21.8	-26.9	-21.1	-6.4	-9.3	-14.1	-9.8	-5.5	**-13.0**
miR-17-5p/20/93.mr/106/519.d	**AAAGUGC**	-7.1	-11.9	-8.5	-21.4	-22.6	-17.5	-6.3	-6.1	-13.4	-12.1	-3.7	**-11.9**
miR-200bc/429	**AAUACUG**	4.7	-6.4	-6.2	-27.3	-22.9	-24.2	-2.6	-3.2	-4.6	-4.1	-6.3	**-9.4**
miR-30a/30a-5p/30b/30b-5p/30cde/384-5p	**GUAAACA**	-7.1	-9.7	-5.8	-26.2	-23.7	-19.9	-2.3	-4.2	-4.7	-3.1	3.6	**-9.4**
miR-19	**GUGCAAA**	-6.5	-9.9	-8.6	-20.9	-16.3	-22.4	-2.9	-3.7	-5.0	-9.6	3.3	**-9.3**
miR-130/301	**AGUGCAA**	-6.3	-7.9	-8.7	-21.7	-19.7	-18.9	-1.4	-4.4	-3.9	-5.6	-3.4	**-9.2**
miR-106/302	**AAGUGCU**	-6.0	-4.0	-5.4	-14.9	-18.8	-14.6	-4.2	-4.6	-11.5	-7.2	-3.7	**-8.6**
miR-340/340-5p	**UAUAAAG**	5.6	-10.2	-8.6	-25.2	-24.1	-28.8	3.2	5.7	4.0	-5.6	-7.4	**-8.3**
miR-218	**UGUGCUU**	-2.5	-8.7	-6.1	-18.7	-25.6	-28.0	2.1	-3.6	-2.2	-1.5	4.1	**-8.3**
miR-182	**UUGGCAA**	-6.0	-7.6	-6.7	-17.0	-19.2	-19.5	-5.6	-2.8	-7.7	-5.4	6.7	**-8.2**
miR-338-5p	**ACAAUAU**	-4.4	-5.7	-10.4	-17.0	-20.5	-19.7	2.0	4.3	-4.7	-4.3	-8.3	**-8.1**
miR-96/1271	**UUGGCAC**	-6.1	-13.0	-10.0	-16.8	-18.8	-16.3	-4.8	3.4	-7.1	-4.2	7.1	**-7.9**
miR-590/590-3p	**AAUUUUA**	-5.1	-6.2	-6.5	-21.0	-15.7	-21.1	3.0	3.4	-4.0	-3.1	-8.7	**-7.7**
miR-181	**ACAUUCA**	-4.6	-5.1	-4.6	-19.2	-20.2	-20.5	3.9	3.4	-4.0	-4.7	-6.7	**-7.5**
miR-25/32/92/92ab/363/367	**AUUGCAC**	-4.8	-6.2	-7.4	-14.0	-14.4	-15.6	-2.3	-4.8	-5.9	-4.1	-1.2	**-7.3**
miR-302ac/520f	**AGUGCUU**	-4.1	-3.5	-3.1	-17.0	-14.9	-13.5	-2.0	-4.3	-5.5	-7.4	-2.9	**-7.1**
miR-495/1192	**AACAAAC**	-4.0	-4.4	-7.3	-22.7	-21.5	-22.1	6.0	6.1	6.1	1.7	-13.0	**-6.8**
miR-142-5p	**AUAAAGU**	-1.7	-6.0	-5.0	-14.7	-16.8	-12.8	-1.5	-4.4	-3.3	-4.9	-3.0	**-6.7**
miR-9	**CUUUGGU**	-5.8	-7.7	-11.2	-11.1	-10.1	-8.9	-6.1	-5.6	-9.6	-4.1	10.3	**-6.3**
miR-133	**UUGGUCC**	-3.9	-8.6	-7.4	-12.5	-15.9	-12.6	-2.5	-4.6	-3.5	-2.0	4.4	**-6.3**
miR-204/211	**UCCCUUU**	-3.9	-5.5	-8.6	-12.7	-14.1	-14.3	-2.4	-4.8	-3.7	-1.6	2.6	**-6.3**
miR-410	**AUAUAAC**	-4.8	-7.9	-4.3	-17.7	-12.1	-16.8	-2.5	4.5	-4.4	-4.1	1.9	**-6.2**
miR-145	**UCCAGUU**	-5.1	-4.3	-3.0	-13.8	-14.1	-18.5	-2.1	-2.8	-3.9	-3.3	3.1	**-6.1**
miR-141/200a	**AACACUG**	-2.9	-7.5	-4.8	-15.7	-17.7	-24.9	3.2	5.6	3.1	-1.6	-3.6	**-6.1**
miR-137	**UAUUGCU**	-4.2	-4.4	-4.7	-15.1	-18.7	-17.6	1.4	2.9	-5.3	-3.2	2.6	**-6.0**
miR-186	**AAAGAAU**	-5.4	-3.0	-4.1	-16.0	-12.9	-17.6	3.9	3.1	-2.6	-1.2	-8.1	**-5.8**
miR-144	**ACAGUAU**	-3.9	-5.3	-5.1	-17.1	-15.8	-18.6	4.4	3.9	-2.7	2.1	-4.7	**-5.7**
miR-323/323-3p	**ACAUUAC**	4.4	-4.6	-3.8	-21.1	-16.7	-14.3	2.8	-5.0	-2.2	2.6	-4.0	**-5.6**
miR-369/369-3p	**AUAAUAC**	-2.4	-3.3	-5.2	-14.0	-15.0	-15.1	-1.1	0.9	-2.3	-1.9	-2.3	**-5.6**
miR-203	**UGAAAUG**	3.0	-5.1	-5.0	-18.7	-18.5	-20.5	5.0	3.6	3.6	-2.8	-3.7	**-5.4**
miR-512-3p/1186	**AGUGCUG**	-3.6	-2.5	-4.1	-11.8	-11.7	-12.6	-1.3	-1.3	-4.4	-2.8	-2.2	**-5.3**
miR-27ab	**UCACAGU**	-3.8	-8.6	-4.4	-15.0	-15.9	-17.3	3.9	5.0	-3.5	-1.5	3.8	**-5.2**
miR-300	**AUACAAG**	10.5	-3.7	-4.3	-22.7	-15.4	-21.0	6.2	9.3	-3.3	-2.5	-9.0	**-5.1**
miR-101	**ACAGUAC**	-4.7	-3.8	-4.1	-17.1	-15.2	-20.0	4.6	6.5	2.2	1.7	-5.9	**-5.1**

miR-543	**AACAUUC**	-3.9	-2.5	-4.2	-19.7	-16.2	-19.5	5.8	3.9	3.9	2.1	-3.8	**-4.9**
miR-183	**AUGGCAC**	-3.4	-5.6	-5.7	-11.4	-18.3	-14.4	2.3	3.4	-1.6	-1.1	2.0	**-4.9**
miR-448	**UGCAUAU**	-3.3	-8.3	-7.0	-12.4	-11.4	-12.9	1.6	4.4	-2.6	-2.9	3.0	**-4.7**
miR-223	**GUCAGUU**	-4.6	-3.4	-2.6	-8.2	-12.4	-12.3	1.3	-1.5	-2.7	-1.6	-3.4	**-4.7**
miR-377	**UCACACA**	-4.3	-2.7	-3.2	-11.4	-9.5	-17.0	-1.2	2.0	-0.8	1.6	-4.3	**-4.6**
miR-1/206	**GGAAUGU**	3.7	-7.2	-4.9	-11.8	-11.1	-16.4	-2.3	2.8	-3.2	-2.1	2.8	**-4.5**
miR-135	**AUGGCUU**	3.3	-6.3	-4.0	-11.4	-12.7	-15.4	-1.4	5.7	-3.6	-1.8	-2.2	**-4.5**
miR-374/374ab	**UAUAAUA**	-2.4	-2.1	-3.2	-10.2	-9.1	-10.6	-1.6	-1.3	-3.0	-2.3	-3.9	**-4.5**
miR-23ab	**UCACAUU**	-4.4	-2.5	-2.2	-16.2	-14.7	-16.9	4.5	5.7	3.3	-1.7	-4.3	**-4.5**
miR-129/129-5p	**UUUUUGC**	-6.6	-4.1	-4.9	-11.7	-14.1	-15.3	2.3	4.3	3.2	3.5	-5.8	**-4.5**
miR-142-3p	**GUAGUGU**	-3.4	-6.7	-5.9	-8.6	-7.7	-10.9	-1.2	-2.5	-2.7	-1.9	2.4	**-4.5**
miR-153	**UGCAUAG**	-5.2	-5.7	-4.0	-11.8	-9.9	-12.8	-1.3	5.0	-2.7	-2.0	2.5	**-4.4**
miR-384/384-3p	**UUCCUAG**	-3.0	-4.3	-4.2	-8.7	-11.3	-8.7	1.2	-4.8	1.1	-0.8	-3.2	**-4.3**
miR-199/199-3p	**CAGUAGU**	-5.4	-1.8	-1.2	-13.2	-10.8	-11.6	-1.8	2.8	-4.0	-2.3	2.6	**-4.2**
miR-103/107	**GCAGCAU**	-2.2	-4.7	-2.5	-13.0	-10.5	-11.2	1.4	4.4	-3.1	-2.2	-2.9	**-4.2**
miR-568	**UGUAUAA**	-3.4	-3.6	-2.9	-9.8	-12.4	-9.2	-1.5	1.7	-3.8	-2.1	1.4	**-4.1**
miR-539	**GAGAAAU**	-4.2	-2.2	-1.3	-12.2	-11.8	-13.4	2.9	-2.8	-2.1	-1.1	2.6	**-4.1**
miR-26ab/1297	**UCAAGUA**	4.8	-4.0	-3.8	-16.1	-19.1	-21.2	4.9	7.6	2.7	2.8	-3.2	**-4.1**
miR-219/219-5p	**GAUUGUC**	2.6	-3.7	-3.4	-8.5	-9.5	-13.6	-1.6	-0.9	-1.5	-1.6	-1.7	**-3.9**
miR-494	**GAAACAU**	2.1	-2.9	-3.0	-11.9	-7.3	-10.1	2.9	-4.2	-4.0	-1.9	-2.8	**-3.9**
miR-128	**CACAGUG**	-3.5	-6.1	-4.2	-12.2	-12.5	-14.6	3.0	4.2	-2.8	2.0	4.0	**-3.9**
miR-15/16/195/424/497	**AGCAGCA**	4.3	-8.0	-8.8	-15.0	-15.2	-15.0	4.0	5.7	3.2	1.8	3.3	**-3.6**
miR-18ab	**AAGGUGC**	-2.0	-1.3	-2.0	-8.7	-6.9	-8.1	-3.2	-1.5	-4.0	-3.3	2.1	**-3.5**
miR-409-3p	**AAUGUUG**	-1.9	-4.4	-4.9	-9.0	-6.7	-11.5	1.0	3.3	3.1	-3.0	-4.2	**-3.5**
miR-376c	**ACAUAGA**	-3.1	-4.8	-5.6	-10.2	-6.6	-7.2	0.8	0.9	-1.4	-1.4	0.8	**-3.4**
miR-31	**GGCAAGA**	-3.4	-2.2	-3.5	-10.8	-10.5	-10.0	2.3	-1.1	2.8	-1.5	0.3	**-3.4**
miR-194	**GUAACAG**	3.2	-2.8	-2.6	-13.0	-13.3	-14.3	2.2	5.3	1.7	1.6	-4.3	**-3.3**
miR-138	**GCUGGUG**	2.7	-3.3	-5.6	-7.8	-11.3	-9.6	-1.3	5.5	-1.4	-1.3	-2.3	**-3.3**
miR-34a/34b-5p/34c/34c-5p/449/449abc/699	**GGCAGUG**	-2.3	-5.1	-5.7	-8.3	-10.5	-10.7	1.1	3.2	-1.2	2.1	1.9	**-3.2**
miR-409-5p	**GGUUACC**	-1.3	-1.3	-3.2	-8.5	-9.1	-11.9	-1.3	2.5	-1.7	-1.2	1.5	**-3.2**
miR-148/152	**CAGUGCA**	-1.8	-2.9	-3.1	-12.7	-9.6	-13.5	2.8	6.1	3.0	-1.2	-1.6	**-3.1**
miR-155	**UAAUGCU**	-2.2	-2.5	-3.5	-8.4	-6.3	-10.6	1.8	2.3	-1.0	-1.8	-2.0	**-3.1**
miR-140/140-5p/876-3p	**AGUGGUU**	-2.4	-2.8	-4.9	-7.7	-5.5	-6.0	-0.9	-1.0	-3.0	-2.6	3.3	**-3.0**
miR-375	**UUGUUCG**	-2.8	-3.8	-2.3	-6.5	-6.8	-5.8	1.7	-2.3	-2.0	-1.4	-1.5	**-3.0**
miR-653	**UGUUGAA**	-1.3	-1.9	-2.3	-9.6	-6.1	-8.8	-0.9	0.9	0.7	-2.5	-1.6	**-3.0**
miR-7/7ab	**GGAAGAC**	-2.2	-2.1	-3.8	-5.9	-5.5	-8.7	1.1	-2.0	-5.1	-1.7	2.9	**-3.0**
miR-221/222	**GCUACAU**	-1.6	-4.6	-4.8	-10.2	-12.5	-9.1	4.4	3.4	4.0	2.3	-4.4	**-3.0**
miR-365	**AAUGCCC**	-3.4	-1.2	-1.4	-5.5	-3.8	-8.3	-2.4	-1.9	-3.3	-3.5	2.3	**-2.9**
miR-592/599	**UUGUGUC**	-1.1	-0.9	-2.3	-7.2	-6.0	-10.4	-1.9	2.2	-1.6	-1.5	-1.4	**-2.9**
miR-129-3p	**AGCCCUU**	2.3	-1.8	-2.2	-10.6	-12.6	-11.2	1.3	-1.3	2.6	1.0	0.9	**-2.9**
miR-320/320abcd	**AAAGCUG**	8.1	-3.6	-4.2	-16.5	-15.4	-18.4	4.0	2.9	4.9	4.5	2.3	**-2.9**

miR-143	**GAGAUGA**	-4.1	-2.6	-2.1	-8.2	-10.4	-7.2	-0.6	2.3	-1.1	1.3	1.7	**-2.8**
miR-29abc	**AGCACCA**	7.7	-9.9	-6.2	-9.7	-10.3	-11.3	-4.3	3.1	3.1	-1.0	8.1	**-2.8**
miR-33/33ab	**UGCAUUG**	-1.2	-1.6	-1.1	-7.2	-5.7	-11.7	1.0	2.0	-1.9	-1.4	-1.7	**-2.8**
miR-344-5p/484	**CAGGCUC**	-2.5	-1.4	-5.3	-6.3	-8.7	-7.3	-1.2	2.2	-1.9	1.0	2.8	**-2.6**
miR-346	**GUCUGCC**	-1.7	-2.6	-1.8	-5.9	-4.4	-8.2	-1.3	1.0	-0.7	-1.7	-1.2	**-2.6**
miR-216/216b	**AAUCUCU**	-1.7	-4.9	-3.5	-4.7	-3.9	-4.6	-2.3	-1.6	-2.1	-1.0	1.9	**-2.6**
miR-335/335-5p	**CAAGAGC**	-0.8	-2.5	-4.3	-4.3	-4.0	-7.5	-1.2	2.5	-2.0	-3.0	-1.2	**-2.6**
miR-544	**UUCUGCA**	-2.5	-2.5	-3.3	-8.7	-5.2	-8.7	2.9	2.2	2.3	-1.0	-3.2	**-2.5**
miR-192/215	**UGACCUA**	0.9	-6.0	-5.4	-3.7	-3.1	-4.2	-1.1	-1.1	-1.1	-3.2	0.8	**-2.5**
miR-876-5p	**GGAUUUC**	-1.2	-1.5	-3.8	-4.7	-5.6	-8.5	1.4	-1.6	-1.3	-1.3	1.3	**-2.4**
miR-488	**UGAAAGG**	-3.4	-2.4	1.4	-6.0	-5.1	-7.6	1.6	1.3	-1.4	-2.4	-2.5	**-2.4**
miR-290-5p/292-5p/371-5p	**CUCAAAC**	-3.1	-2.1	-3.6	-8.4	-8.3	-11.0	4.0	3.9	4.3	2.2	-3.9	**-2.4**
miR-485/485-5p	**GAGGCUG**	-1.9	-1.8	-3.1	-3.5	-3.0	-5.0	-3.2	-2.7	-3.3	-1.0	2.5	**-2.4**
miR-139-5p	**CUACAGU**	4.0	1.6	-1.8	-11.8	-9.3	-15.8	4.1	-1.4	3.8	2.7	-1.7	**-2.3**
miR-205	**CCUUCAU**	3.2	-3.1	-4.2	-8.4	-9.2	-9.0	1.3	3.3	2.5	1.0	-2.8	**-2.3**
miR-338/338-3p	**CCAGCAU**	-2.0	-1.9	-1.6	-5.9	-7.6	-8.9	0.8	1.7	1.8	0.9	-2.0	**-2.3**
miR-214/761	**CAGCAGG**	-2.9	-1.9	-3.3	-5.2	-7.9	-11.2	0.5	3.3	2.0	0.8	2.2	**-2.1**
miR-216/216a	**AAUCUCA**	-3.2	-3.2	-2.6	-5.0	-4.8	-5.4	0.7	1.5	-1.3	0.7	-1.0	**-2.1**
miR-763/1207-3p	**CAGCUGG**	-1.4	-2.6	-5.4	-2.4	-3.6	-4.7	-2.3	-1.2	-2.3	-1.0	3.6	**-2.1**
miR-709/1827	**GAGGCAG**	1.0	-3.4	-4.2	-6.7	-6.2	-6.6	-1.9	1.2	-0.7	-1.0	5.6	**-2.1**
miR-425/489	**AUGACAC**	1.7	-1.3	-1.8	-4.5	-3.2	-7.1	-2.5	2.3	-3.6	-0.9	-1.9	**-2.1**
miR-150	**CUCCCAA**	-1.7	-4.6	-2.7	-5.0	-4.4	-5.8	-1.1	2.0	-1.9	-0.7	3.0	**-2.1**
miR-501/501-5p	**AUCCUUU**	-2.5	-1.4	-2.0	-6.2	-5.5	-6.4	1.9	-2.1	2.5	0.9	-1.8	**-2.0**
miR-190	**GAUAUGU**	-2.0	-2.7	-1.2	-9.6	-3.7	-4.9	0.5	1.6	2.6	1.8	-4.7	**-2.0**
miR-542/542-3p	**GUGACAG**	1.8	-2.6	-2.0	-4.7	-4.5	-7.2	1.4	-2.4	-2.3	-1.6	2.6	**-2.0**
miR-151-3p	**UAGACUG**	-2.6	-1.8	-1.3	-4.3	-6.1	-7.1	1.5	2.3	0.7	0.5	-2.6	**-1.9**
miR-361/361-5p	**UAUCAGA**	-2.4	-2.0	-1.5	-4.3	-3.8	-6.3	0.8	0.5	-0.7	-0.4	-0.9	**-1.9**
miR-370	**CCUGCUG**	-1.3	-1.8	-2.3	-2.5	-3.5	-5.8	-1.5	-3.6	1.4	-1.9	2.3	**-1.9**
miR-505.hm	**GUCAACA**	1.6	-2.2	2.7	-6.8	-3.9	-4.6	-1.2	-1.0	-2.2	-0.6	-1.7	**-1.8**
miR-431	**GUCUUGC**	-1.7	-1.3	-2.8	-5.0	-3.2	-5.0	-1.5	1.1	2.0	-1.0	-1.4	**-1.8**
miR-877	**UAGAGGA**	0.9	-1.2	-2.1	-4.1	-2.8	-4.7	-1.4	0.3	-2.3	-1.6	-0.8	**-1.8**
miR-217	**ACUGCAU**	4.2	0.9	-1.5	-8.9	-9.4	-12.4	3.8	3.6	1.9	0.9	-2.6	**-1.8**
miR-873	**CAGGAAC**	-2.5	-1.3	-1.4	-6.3	-7.6	-9.9	1.9	3.6	1.4	1.4	1.4	**-1.7**
miR-496	**GAGUAUU**	1.2	-2.5	-1.8	-5.2	-3.5	-4.9	-1.7	-0.5	-1.2	-0.8	1.7	**-1.7**
miR-379	**GGUAGAC**	-1.4	-3.5	-2.9	-2.0	-1.9	-2.6	0.6	-1.0	-0.8	-1.5	-1.9	**-1.7**
miR-671-5p	**GGAAGCC**	-0.9	-3.6	-1.1	-1.6	-2.9	-3.5	-4.1	1.4	-3.4	-2.9	3.9	**-1.7**
miR-329/362-3p	**ACACACC**	-1.5	1.9	-2.6	-6.5	-3.8	-8.0	1.6	1.8	-0.9	1.9	-2.2	**-1.7**
miR-499/499-5p	**UAAGACU**	-1.8	-2.0	-2.1	-5.7	-5.9	-8.0	3.1	2.5	1.7	2.8	-2.7	**-1.7**
miR-21/590-5p	**AGCUUAU**	3.3	1.1	-2.4	-3.7	-3.7	-6.0	-1.4	1.7	-2.0	-1.6	-3.4	**-1.7**
miR-122	**GGAGUGU**	-1.7	-3.8	-4.3	-2.7	-2.9	-3.6	-2.3	1.7	-2.1	0.4	3.3	**-1.6**
miR-376/376ab/376b-3p	**UCAUAGA**	2.4	-4.5	-5.9	-3.0	-2.4	-1.7	1.7	-1.6	-3.3	-1.1	1.4	**-1.6**
miR-149	**CUGGCUC**	-1.4	-4.1	-2.4	-3.3	-3.9	-7.4	1.0	1.9	-0.9	-0.9	3.5	**-1.6**

miR-508-5p	**ACUCCAG**	1.6	-2.6	-1.2	-5.3	-7.5	-8.7	1.8	1.0	0.9	1.1	1.3	**-1.6**
miR-500/501-3p/502/502-3p	**AUGCACC**	2.2	-1.3	-1.5	-6.9	-6.2	-10.8	2.5	1.8	2.5	0.7	-0.5	**-1.6**
miR-486/486-5p	**CCUGUAC**	0.7	-1.5	-1.5	-6.1	-6.1	-5.0	0.7	1.9	0.6	0.8	-1.9	**-1.6**
miR-199/199-5p	**CCAGUGU**	3.1	-2.0	-2.9	-7.1	-6.6	-6.1	1.3	3.5	0.9	0.9	-1.9	**-1.5**
miR-881/892a	**ACUGUGU**	1.1	-1.3	-1.4	-4.3	-3.7	-4.5	1.3	1.1	0.9	-1.7	-3.6	**-1.5**
miR-654-3p	**AUGUCUG**	-3.2	-0.9	0.9	-5.1	-6.8	-5.8	3.3	1.3	2.6	1.6	-4.2	**-1.5**
miR-581/669d	**CUUGUGU**	-1.3	-1.3	-3.8	-2.7	-2.2	-2.0	-1.1	-1.9	2.1	0.5	-2.2	**-1.4**
miR-326/330/330-5p	**CUCUGGG**	2.1	-3.6	-2.7	-2.7	-5.2	-5.9	-1.0	3.0	-1.1	-0.7	2.5	**-1.4**
miR-185/882	**GGAGAGA**	-1.8	-2.5	-3.8	-2.2	-1.9	-3.6	-0.5	2.5	-1.6	-1.3	1.4	**-1.4**
miR-503	**AGCAGCG**	2.6	-2.1	-4.9	-7.0	-6.7	-7.4	2.3	4.3	5.0	1.9	-2.5	**-1.3**
miR-1224/1224-5p	**UGAGGAC**	-2.0	-1.6	-2.2	-2.7	-3.2	-5.0	0.9	2.1	-2.6	-2.0	4.0	**-1.3**
miR-758	**UUGUGAC**	-0.9	-4.7	-0.8	-5.2	-3.0	-4.6	0.7	2.9	1.5	-2.3	2.0	**-1.3**
miR-532/532-5p	**AUGCCUU**	2.2	-1.9	-1.8	-4.6	-4.1	-4.5	-1.1	1.6	1.5	0.8	-2.3	**-1.3**
miR-297/297a/297b-5p/297c	**UGUAUGU**	-2.3	-1.9	-1.5	-4.6	-2.1	-6.3	1.5	2.8	1.7	0.5	-1.9	**-1.3**
miR-146	**GAGAACU**	-1.2	-1.4	-1.3	-4.2	-3.4	-3.2	-0.8	1.8	0.8	0.7	-1.4	**-1.2**
miR-668	**GUCACUC**	1.4	-2.6	-2.4	-2.8	-3.8	-2.9	1.1	-1.1	-0.8	-1.1	1.3	**-1.2**
miR-10	**ACCCUGU**	3.0	-2.4	-4.2	-4.6	-5.6	-4.4	1.8	1.7	1.3	3.3	-3.4	**-1.2**
miR-132/212	**AACAGUC**	2.3	-1.6	-1.2	-8.0	-6.5	-8.0	2.3	2.0	2.7	4.3	-1.8	**-1.2**
miR-328	**UGGCCCU**	-1.2	-2.7	-4.6	-2.4	-3.0	-1.7	-0.7	0.4	-1.3	-0.9	4.9	**-1.2**
miR-154	**AGGUUAU**	2.1	-1.1	-2.9	-3.6	-2.8	-3.6	0.9	2.3	-1.5	0.7	-2.5	**-1.1**
miR-760/1842	**GGCUCUG**	2.8	-2.4	-2.1	-3.7	-4.8	-2.8	-1.0	1.6	-1.4	-0.4	2.4	**-1.1**
miR-22	**AGCUGCC**	1.6	-2.1	-2.4	-5.8	-6.2	-9.0	1.3	4.5	3.1	1.3	2.7	**-1.0**
miR-193ab	**ACUGGCC**	-3.1	1.4	1.0	-4.6	-6.7	-7.1	4.5	2.7	2.6	1.2	-2.8	**-1.0**
miR-324-5p	**GCAUCCC**	-1.0	-1.0	-2.9	-5.2	-4.4	-5.3	2.5	2.5	1.6	1.6	0.9	**-1.0**
miR-1197	**AGGACAC**	-1.4	-1.5	-1.0	-6.2	-4.6	-7.7	1.8	3.0	3.2	1.9	2.3	**-0.9**
miR-296/296-3p	**AGGGUUG**	-0.9	-1.8	-2.0	-3.2	-2.5	-2.9	1.4	1.5	-1.7	1.2	1.0	**-0.9**
miR-665	**CCAGGAG**	-1.1	-1.8	-2.1	-3.9	-2.9	-6.1	1.1	4.2	-1.3	1.3	3.1	**-0.9**
miR-202/202-3p	**GAGGUAU**	2.0	-4.9	-2.1	-6.8	-5.3	-5.7	1.7	5.0	2.5	2.5	1.8	**-0.8**
miR-455/455-3p	**CAGUCCA**	2.7	-1.4	-0.7	-5.4	-6.3	-6.5	2.7	4.2	2.6	2.2	-3.0	**-0.8**
miR-299/299-3p	**AUGUGGG**	-1.2	-1.8	-1.0	-2.6	-1.5	-2.9	0.8	1.0	0.7	-1.1	0.9	**-0.8**
miR-136	**CUCCAUU**	1.9	-2.9	-2.7	-2.1	-3.1	-7.7	1.8	0.7	1.6	1.2	2.8	**-0.8**
miR-433	**UCAUGAU**	-3.7	-0.6	-0.9	-5.8	-4.6	-2.9	3.7	2.3	3.3	-1.4	2.1	**-0.8**
miR-875-5p	**AUACCUC**	0.8	1.1	-0.8	-3.2	-2.2	-3.1	1.2	1.1	-0.6	-1.1	-1.6	**-0.8**
miR-362-5p	**AUCCUUG**	-1.3	-0.7	-1.7	-1.8	-2.3	-3.7	1.1	2.8	2.4	-0.6	-2.4	**-0.7**
miR-125/351	**CCCUGAG**	-3.7	-1.3	-2.1	-5.9	-7.6	-4.8	1.0	5.3	2.1	1.6	7.3	**-0.7**
miR-34b-3p/34c-3p	**AUCACUA**	1.9	0.9	2.0	-1.3	-3.3	-5.8	1.6	-2.6	2.9	-1.1	-3.2	**-0.7**
miR-191	**AACGGAA**	-0.5	-2.4	-1.2	-2.0	-1.1	-1.3	0.8	0.7	-0.5	-0.7	0.9	**-0.7**
miR-574-5p	**GAGUGUG**	-2.8	-1.1	-1.3	-3.3	-1.8	-2.6	1.4	2.8	1.6	1.1	-1.1	**-0.7**
miR-134	**GUGACUG**	-1.3	2.0	2.9	-1.5	-2.5	-1.6	-1.6	1.3	-1.1	-2.1	-1.5	**-0.6**
miR-208/208ab	**UAAGACG**	-1.6	1.4	-0.7	-3.7	-4.2	-5.7	3.9	2.0	1.6	3.2	-2.9	**-0.6**
miR-342/342-3p	**CUCACAC**	1.6	-1.5	-2.4	-3.3	-2.3	-6.5	1.3	4.1	1.0	2.9	-1.8	**-0.6**
miR-342-5p	**GGGGUGC**	-3.5	-1.4	-1.9	-1.5	-1.2	-2.2	-0.4	0.7	-1.3	0.5	5.4	**-0.6**

miR-127	**CGGAUCC**	-0.7	1.0	-0.7	-1.1	-1.1	-0.7	-0.7	-0.1	-1.6	-0.9	-0.1	**-0.6**
miR-296-5p	**GGGCCCC**	-0.7	-1.5	-2.9	-2.0	-0.8	-1.4	-1.4	0.5	-0.6	0.5	3.8	**-0.6**
miR-187	**CGUGUCU**	-1.4	-0.7	-0.2	-1.2	-1.6	-1.7	-0.4	1.3	-0.6	-0.6	0.7	**-0.6**
miR-378/422a	**CUGGACU**	2.0	-1.2	-1.6	-2.5	-4.1	-3.8	1.3	3.2	0.9	1.1	-1.5	**-0.6**
miR-339-5p	**CCCUGUC**	1.3	-2.1	-1.5	-4.5	-3.6	-4.5	1.7	3.7	2.8	1.8	-0.8	**-0.5**
let-7/98	**GAGGUAG**	3.4	-3.6	-3.3	-5.7	-4.0	-5.1	0.9	5.3	2.2	2.5	1.9	**-0.5**
miR-582-3p/1267	**CUGUUGA**	1.7	-1.1	-2.5	-2.0	-1.5	-4.9	1.7	0.4	0.8	0.7	1.5	**-0.5**
miR-224	**AAGUCAC**	-4.2	1.8	4.3	-3.5	-2.4	-5.1	2.6	1.7	1.2	1.1	-2.9	**-0.5**
miR-210	**UGUGCGU**	0.2	-0.2	-0.4	-0.8	-1.0	-0.6	-0.9	-0.8	-1.7	1.2	-0.2	**-0.5**
miR-331-5p	**UAGGUAU**	0.6	-1.4	-0.5	-2.3	-1.7	-1.4	0.7	0.5	0.5	0.6	-0.6	**-0.5**
miR-126/126-3p	**CGUACCG**	1.1	-0.6	0.4	-1.0	-2.3	-1.8	0.3	-0.4	-0.5	-0.6	0.5	**-0.4**
miR-504	**GACCCUG**	-0.8	-1.7	-2.4	-2.2	-2.2	-1.3	1.3	0.9	1.5	1.4	0.7	**-0.4**
miR-487/487b	**AUCGUAC**	-0.4	-0.8	-0.4	-0.6	-1.6	-1.8	-0.3	1.1	0.0	0.1	0.2	**-0.4**
miR-421	**UCAACAG**	1.7	3.4	2.7	-7.7	-5.3	-8.4	3.9	4.9	5.3	1.8	-6.8	**-0.4**
miR-675/675-5p	**GGUGCGG**	-0.6	-0.3	-1.7	-1.3	-1.1	-0.5	-0.4	0.6	-0.1	1.2	-0.1	**-0.4**
miR-411	**AGUAGAC**	1.3	-2.2	-0.3	-0.7	-3.2	-4.4	2.8	1.5	1.2	1.0	-1.1	**-0.4**
miR-652	**AUGGCGC**	-0.7	-0.5	-0.5	-0.4	-0.3	-0.7	-0.6	0.9	-0.6	0.6	-1.3	**-0.4**
miR-744	**GCGGGGC**	0.4	-0.3	-1.4	-0.6	-0.4	-0.7	-0.8	-0.3	-0.8	0.9	0.1	**-0.4**
miR-493	**GAAGGUC**	0.5	1.3	0.6	-1.4	-1.4	-4.1	0.8	0.2	-1.2	-0.5	1.3	**-0.3**
miR-874	**UGCCCUG**	-0.7	-0.6	0.9	-1.4	-2.4	-1.6	-0.9	1.7	-1.6	-1.1	4.5	**-0.3**
miR-196ab	**AGGUAGU**	5.6	2.4	-3.5	-4.1	-3.8	-7.8	3.2	2.6	1.4	2.5	-1.7	**-0.3**
miR-323-5p	**GGUGGUC**	1.5	-0.7	-1.5	-0.9	-0.7	-2.8	0.7	-1.6	1.5	0.8	0.7	**-0.3**
miR-382	**AAGUUGU**	0.8	1.2	1.9	-5.2	-2.9	-4.3	1.7	4.6	2.0	-1.2	-1.5	**-0.3**
miR-147/147b	**UGUGCGG**	0.2	-0.2	-0.2	-1.1	-1.1	-0.6	-0.6	-0.5	-0.3	1.1	0.4	**-0.3**
miR-450a/450a-5p	**UUUGCGA**	-0.2	-0.3	-0.4	-0.5	-1.3	-0.9	0.4	0.6	0.6	0.3	-0.7	**-0.2**
miR-615-5p	**GGGGUCC**	0.0	-0.3	-2.1	-0.2	-0.8	-1.1	-0.5	0.7	0.4	0.3	1.4	**-0.2**
miR-125a-3p	**CAGGUGA**	3.2	-1.0	0.8	-2.5	-2.6	-4.2	1.8	0.8	-1.3	1.3	1.6	**-0.2**
miR-188/188-5p	**AUCCCUU**	1.0	0.8	2.1	-3.4	-3.7	-4.8	3.6	3.0	1.9	-1.1	-1.1	**-0.1**
miR-151	**CGAGGAG**	-0.6	-0.1	-0.3	-0.3	-0.1	-0.5	0.3	-0.3	0.2	0.4	-0.1	**-0.1**
miR-184	**GGACGGA**	-0.6	0.7	0.7	-1.2	-1.4	-1.5	-1.0	0.5	1.1	0.4	1.1	**-0.1**
miR-28/28-5p/708	**AGGAGCU**	1.2	-1.2	-1.4	-1.4	-1.6	-2.2	0.7	1.7	1.1	0.6	1.3	**-0.1**
miR-383	**GAUCAGA**	1.0	0.8	-1.5	-2.9	-1.7	-3.4	-0.9	4.2	1.8	0.5	2.2	**0.0**
miR-369-5p	**GAUCGAC**	-1.4	0.3	-0.8	-0.3	0.5	0.2	-0.2	1.0	0.3	0.8	-0.3	**0.0**
miR-339-3p	**GAGCGCC**	0.6	0.5	0.4	1.1	0.7	0.3	-1.4	0.2	-2.1	-1.9	2.2	**0.0**
miR-197	**UCACCAC**	4.0	-1.8	-1.6	-3.6	-4.8	-5.9	3.3	2.2	3.5	3.0	2.0	**0.0**
miR-483/483-5p	**AGACGGG**	-0.1	2.4	0.7	-0.8	0.1	-0.3	0.6	-0.4	0.5	-0.5	-1.1	**0.1**
miR-532-3p	**CUCCCAC**	1.5	-0.8	-1.2	-2.1	-1.4	-3.2	0.9	4.3	0.7	0.8	1.3	**0.1**
miR-491/491-5p	**GUGGGGA**	-0.5	-1.1	-1.5	1.0	-0.2	-0.3	-1.5	0.9	-0.6	1.1	3.7	**0.1**
miR-551ab	**CGACCCA**	-0.6	-0.4	-1.3	-0.2	0.4	0.1	0.4	1.6	0.3	1.2	-0.3	**0.1**
miR-331/331-3p	**CCCCUGG**	0.7	0.9	-1.5	-1.0	-0.4	-0.8	-1.0	2.8	-0.3	0.9	0.9	**0.1**
miR-574/574-3p	**ACGCUCA**	0.5	0.3	0.6	0.5	-0.1	0.2	0.4	-1.1	1.1	-0.4	-0.4	**0.1**
miR-615-3p	**CCGAGCC**	-1.0	1.1	1.5	0.8	0.7	-1.3	0.4	0.1	-0.5	0.3	-0.3	**0.2**
miR-188-3p	**UCCCACA**	4.3	0.7	-0.7	-3.8	-1.6	-3.1	2.0	3.9	1.2	1.8	-3.0	**0.2**
miR-598/598-3p	**ACGUCAU**	2.2	-0.8	0.9	-0.8	-0.7	-2.0	0.8	1.1	1.1	1.2	-0.5	**0.2**

miR-99ab/100	**ACCCGUA**	-0.9	-0.7	-1.2	-0.4	-0.6	-0.7	2.2	2.5	0.6	2.5	-0.8	**0.2**
miR-671/671-3p	**CCGGUUC**	-0.4	0.3	-0.2	0.4	0.7	0.7	0.3	0.7	0.6	0.3	-0.3	**0.3**
miR-423a/423-5p	**GAGGGGC**	1.2	1.1	-3.3	-0.6	-0.5	-1.1	-0.9	2.4	-0.8	1.5	4.1	**0.3**
miR-451	**AACCGUU**	0.3	0.8	0.5	-0.4	0.5	-0.2	0.8	1.5	0.2	1.3	-2.0	**0.3**
miR-423/423-3p	**GCUCGGU**	0.2	0.2	0.7	0.4	0.2	0.3	0.3	1.7	0.6	2.0	-0.7	**0.5**
miR-24	**GGCUCAG**	1.0	0.9	-1.3	-3.2	-2.9	-3.7	2.9	4.7	1.3	5.0	1.8	**0.6**
miR-490/490-3p	**AACCUGG**	3.4	1.4	2.3	-2.6	-3.1	-2.9	3.3	2.7	5.0	1.7	-1.9	**0.8**

Values in the table are significance scores (SS) described in Methods. miRNA families are sorted by the mean SS.
