# Supplementary material for: Reprogramming of 3′ Untranslated Regions of mRNAs by Alternative Polyadenylation in Generation of Pluripotent Stem Cells from Different Cell Types
Source: PLoS One. 2009 Dec 23;4(12):e8419. doi: 10.1371/journal.pone.0008419 (PMC2791866; doi:10.1371/journal.pone.0008419)
Supplement: Table S5 — Significant Gene Ontology terms associated with genes in group 1. (0.01 MB PDF) [file pone.0008419.s016.pdf]

Table S5. Significant Gene Ontology terms associated with genes in group 1.

P-value[a]	GO ID, name
3.6E-03	GO:0016567, protein ubiquitination
6.5E-03	GO:0016311, Dephosphorylation
7.9E-03	GO:0006397, mRNA processing
1.2E-02	GO:0006396, RNA processing
3.5E-02	GO:0009968, negative regulation of signal transduction
4.9E-02	GO:0022402, cell cycle process

[a]Based on Fisher's exact test.
